# Atrial nitroso-redox balance and refractoriness following on-pump cardiac surgery: a randomized trial of atorvastatin

**DOI:** 10.1093/cvr/cvaa302

**Published:** 2020-10-24

**Authors:** Raja Jayaram, Michael Jones, Svetlana Reilly, Mark J Crabtree, Nikhil Pal, Nicola Goodfellow, Keshav Nahar, Jillian Simon, Ricardo Carnicer, Ravi DeSilva, Chandana Ratnatunga, Mario Petrou, Rana Sayeed, Andrea Roalfe, Keith M Channon, Yaver Bashir, Timothy Betts, Michael Hill, Barbara Casadei

**Affiliations:** 1 Division of Cardiovascular Medicine, Radcliffe Department of Medicine, University of Oxford, L6, West Wing, Oxford OX3 9DU, UK; 2 Cardiology, Oxford Heart Centre, Oxford University Hospitals NHS Foundation Trust, Oxford, UK; 3 Cardiothoracic Surgery, Oxford Heart Centre, Oxford University Hospitals NHS Foundation Trust, Oxford, UK; 4 Nuffield Department of Primary Care Health Sciences, University of Oxford, Oxford, UK; 5 Clinical Trial Service Unit, Nuffield Department of Population Health, University of Oxford, Oxford, UK

**Keywords:** Atorvastatin, Atrial refractory period, Clinical trial, Ischaemia-Reperfusion Injury, Nitric Oxide Synthase, Oxidant stress, Postoperative Atrial fibrillation

## Abstract

**Aims:**

Systemic inflammation and increased activity of atrial NOX2-containing NADPH oxidases have been associated with the new onset of atrial fibrillation (AF) after cardiac surgery. In addition to lowering LDL-cholesterol, statins exert rapid anti-inflammatory and antioxidant effects, the clinical significance of which remains controversial.

**Methods and results:**

We first assessed the impact of cardiac surgery and cardiopulmonary bypass (CPB) on atrial nitroso-redox balance by measuring NO synthase (NOS) and GTP cyclohydrolase-1 (GCH-1) activity, biopterin content, and superoxide production in paired samples of the right atrial appendage obtained before (PRE) and after CPB and reperfusion (POST) in 116 patients. The effect of perioperative treatment with atorvastatin (80 mg once daily) on these parameters, blood biomarkers, and the post-operative atrial effective refractory period (AERP) was then evaluated in a randomized, double-blind, placebo-controlled study in 80 patients undergoing cardiac surgery on CPB. CPB and reperfusion led to a significant increase in atrial superoxide production (74% CI 71–76%, *n* = 46 paired samples, *P* < 0.0001) and a reduction in atrial tetrahydrobiopterin (BH_4_) (34% CI 33–35%, *n* = 36 paired samples, *P* < 0.01), and in GCH-1 (56% CI 55–58%, *n* = 26 paired samples, *P* < 0.001) and NOS activity (58% CI 52–67%, *n* = 20 paired samples, *P* < 0.001). Perioperative atorvastatin treatment prevented the effect of CPB and reperfusion on all parameters but had no significant effect on the postoperative right AERP, troponin release, or NT-proBNP after cardiac surgery.

**Conclusion:**

Perioperative statin therapy prevents post-reperfusion atrial nitroso-redox imbalance in patients undergoing on-pump cardiac surgery but has no significant impact on postoperative atrial refractoriness, perioperative myocardial injury, or markers of postoperative LV function.

**Clinical Trial Registration:**

https://clinicaltrials.gov/ct2/show/NCT01780740

## Graphical Abstract

**Figure:**
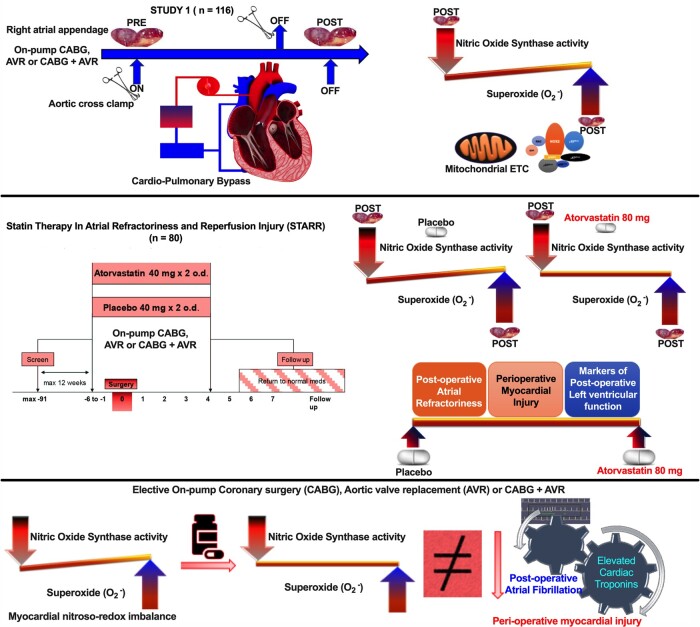

## 1. Introduction

Cardiac surgery is associated with a systemic inflammatory response and a rise in plasma markers of oxidative stress.^1^^,^^2^ Notwithstanding cardioplegia, hearts undergoing surgical procedures on cardiopulmonary bypass (CPB) undergo some degree of ischaemia–reperfusion (I/R) injury.^3^ In isolated hearts, I/R increases the myocardial formation of reactive oxygen species (ROS),^4^^,^^5^ leading to oxidation of the nitric oxide synthase (NOS) co-factor, tetrahydrobiopterin (BH_4_),^6^ and a further increase in ROS formation.^7^ An imbalance between atrial nitric oxide (NO) and ROS production has been associated with the new onset of atrial fibrillation (AF); in particular, the activity of atrial ROS-generating enzymes, such as NOX2-containing NADPH oxidases, is independently associated with postoperative AF in patients undergoing cardiac surgery,^1^^,^^8–10^ whereas knockout of the neuronal NOS isoform increases the probability of AF induction in mice.^11^

Inhibition of 3-Hydroxy-3-methyl glutaryl-coenzyme A reductase by statins has been shown to inhibit myocardial NOX2 activity^8^^,^^12^^,^^13^ and increase vascular NO availability^14^ in humans, and to prevent the shortening of the right atrial effective refractory period (AERP) in dogs in response to rapid atrial pacing.^15^ Meta-analyses of small randomized trials had suggested that perioperative statin treatment may halve the incidence of postoperative AF^16–18^; however, these findings were not confirmed by several recent studies,^19–21^ including a much larger randomized trial,^16^ and by an updated systematic review.^22^

Here, we examined the impact of cardiac surgery and CPB on NOS activity and NOX2-derived superoxide production and biopterin synthesis in samples of the right atrial appendage obtained before (PRE) and after CPB and reperfusion (POST) from 116 patients undergoing first-time elective cardiac surgery. We then assessed whether perioperative statin treatment mitigates the impact of surgery and CPB on the atrial nitroso-redox balance and affects right atrial refractoriness in 80 patients randomized to perioperative atorvastatin (80 mg daily) or placebo (the Statin Therapy in Atrial Refractoriness and Reperfusion Injury Trial or STARR, NCT01780740).

## 2. Methods

### 2.1 Patient population and study protocols

One hundred and sixteen patients in sinus rhythm undergoing their first elective cardiac surgery on CPB (coronary revascularization, aortic valve replacement, or both) in the Department of Cardiothoracic Surgery of the Oxford University Hospitals NHS Trust were recruited in the observational study (Supplementary material online, *Table S1*).

Eighty patients in sinus rhythm (as above) were recruited in a randomized, double-blind, placebo-controlled trial of atorvastatin (80 mg od) (Supplementary material online, *Figure S1B*). Patients were excluded if they were older than 85 years, had a contraindication to statin therapy, or received treatment with antiarrhythmic agents other than beta-adrenergic receptor blockers. Eligible patients were informed about the trial and provided written informed consent. Consenting patients had blood collected for the determination of Troponin I (TnI), the N-terminal fragment of the prohormone brain natriuretic peptide (NT-proBNP), and LDL cholesterol. Any prescribed statin therapy was stopped, and patients were then randomly allocated to receive atorvastatin 80 mg once daily or matching placebo tablets for up to 6 days before surgery and for 5 days thereafter. Myocardial protection in the on-pump procedure was typically achieved by cold blood cardioplegia and moderate systemic hypothermia. Postoperative management was in accordance with standard protocols.

Atorvastatin and matching placebo tablets were donated by Pfizer (UK) and packaged by Catalent Pharma Solutions (UK). The study medications were labelled with sequential numbers, according to a randomization schedule generated at the Clinical Trial Service Unit (CTSU), University of Oxford,

The primary endpoints were the PRE-POST difference in atrial superoxide production and post-operative changes in the right AERP. Secondary endpoints included: atrial NOS activity, GCH1 activity, atrial levels of biopterins, and blood biomarkers.

The local ethics committee approved both studies, and all patients provided written informed consent. For STARR, clinical trial authorization was obtained from the Medicines and Healthcare products Regulatory Agency, UK (Eudra CT ref no: 2009-013228-21) and all investigations conformed to the principles outlined in the Declaration of Helsinki.

### 2.2 Tissue and blood samples

A sample of the right atrial appendage was taken at two time-points: before the insertion of the venous cannula in the right atrium and commencement of cardioplegia (PRE), and soon after venous decannulation and cardiac reperfusion (POST).

In STARR, blood samples were collected at randomization and at 72 h after surgery. Blood samples were processed within 1 h of collection, and plasma/serum aliquots were stored at −80^°^C in bar-coded cryovials. Immunoblots and measurements of atrial superoxide production, NOS activity, atrial biopterins, and GTP-cyclohydrolase 1 (GCH-1) activity were performed as detailed in Supplementary material online.

### 2.3 Measurement of the right AERP

The right AERP was measured daily for 4 days after surgery using a programmed stimulation protocol delivered by the Medtronic Pacing System Analyser 2090 *via* a Medtronic pacemaker (Sensia: SESR01) connected to right atrial epicardial pacing wires that were routinely inserted at the time of surgery. A continuous 12-lead electrocardiogram (ECG) was acquired throughout the protocol to confirm atrial capture and measure AERP accordingly. A conditioning train of 100 stimuli (S1) was delivered at 2 × diastolic threshold followed by pacing at a cycle length of 500, 600, and 700 ms. An atrial extra stimulus (S2) was introduced after every eighth S1 with an initial coupling of 156 ms and with no pauses in the drive train. The atrial capture was defined as either the presence of clearly visible ‘P’ wave on the continuous ECG after S2 or a sinus cycle length of the first return intrinsic beat after S2 (Supplementary material online, *Figure S2*). The coupling interval of the extra stimulus was increased in steps of 16 ms until atrial capture was achieved and continued for another three coupling intervals. All measurements were performed in duplicates. The AERP was defined as the longest S1–S2 coupling interval that failed to result in atrial capture.

### 2.4 Statistical analysis

Data are summarized as mean [standard deviation (SD)], median [interquartile range (IQR)] or geometric mean, and 95% confidence intervals (CIs), as appropriate.

In the observational study, normally distributed variables PRE and POST reperfusion were compared using the paired *t*-test, whereas non-normally distributed data were analysed using the Wilcoxon matched-pairs signed rank test. The POST differences in samples treated with DTT or gp91-ds-tat-peptide vs. corresponding controls were compared by analysis of covariance (ANCOVA) adjusting for PRE measurements.

#### 2.4.1 Trial of perioperative atorvastatin (STARR)

Pilot data indicated that 36 patients per group would be needed to detect a 50% difference in the PRE-POST change atrial superoxide production as evaluated by the area under the 2-OH ethidium curve between treatment groups with 90% statistical power at 5% significance level and 26 patients per group to detect the same difference with 80% power. As studies measuring post-operative right AERP in contemporary patient series are lacking, we derived our sample size based on the findings (15 unit difference, SD of 18.5) reported in a study where simvastatin treatment prevented AERP shortening induced by rapid atrial pacing in dogs.^15^ We calculated that a sample size of 15 patients per arm would be required to detect at least a 20% difference in mean AERP measurements between the treatment groups with 80% power, at 5% significance level.

Only patients who underwent their scheduled cardiac surgery or had at least one of the measurements specified in primary or secondary endpoints were included in the data analysis and tables. AERP measurements obtained in atorvastatin and placebo-treated patients over four post-operative days were subjected to a mixed model linear regression analysis after log transformation of the non-normally distributed data. POST variables and blood biomarkers at 72 h after surgery in STARR were compared by ANCOVA adjusted for baseline value measured at randomization. The Fisher's exact test was used to compare dichotomous variables including postoperative outcomes.

The level of statistical significance was set at 0.05 (two-sided). Statistical analyses were performed using Graph Pad Prism version 7.00 for Mac (Graph Pad Software, San Diego, CA, USA), SPSS Statistical Software (IBM Corp. Released 2017. IBM SPSS Statistics for Mac, Version 25.0. Armonk, NY: IBM Corp) or Stata15.0 (StataCorp, College Station, TX, USA).

## 3. Results

### 3.1 CPB and reperfusion are associated with increased atrial superoxide production and reduced NOS activity

**Figure 1:**
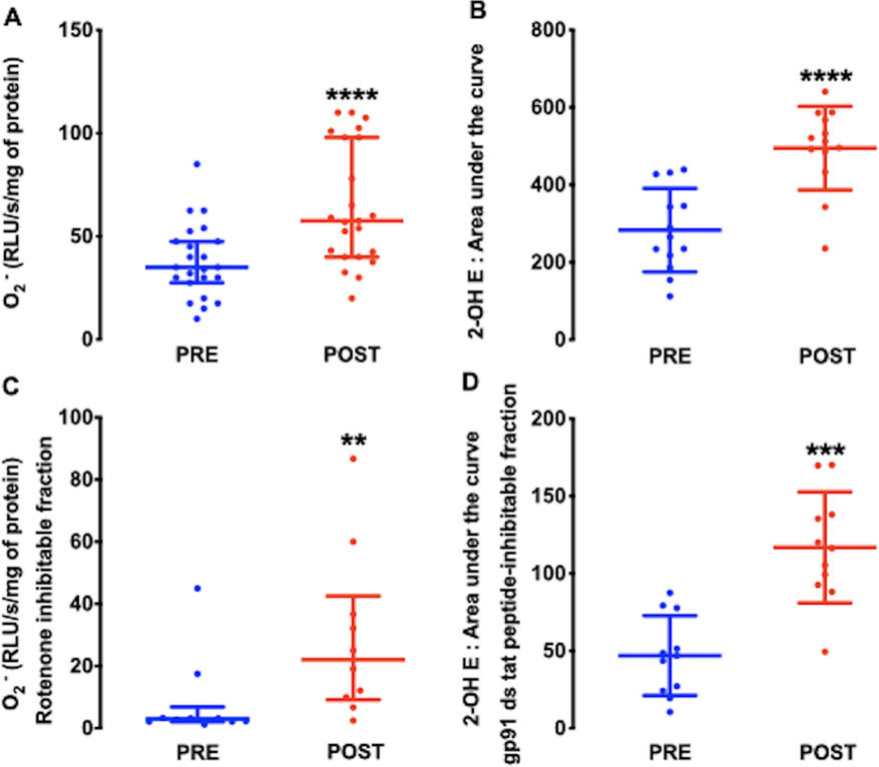
Superoxide ($O_{2}^{-}$) production in right atrial samples obtained before (PRE) and after CPB and reperfusion (POST) measured by lucigenin-enhanced chemiluminescence (*n* = 46 paired samples from 23 patients; *A*) and by HPLC detection of 2-OH ethidium (*n* = 26 paired samples from 13 patients; *B*), respectively. ****P < 0.0001 vs. PRE, by Wilcoxon matched-pairs signed rank test (*A*) or the paired Student’s *t*-test (*B*). The contribution of mitochondrial complex I (*n* = 20 paired samples from 10 patients; *C*) or NOX2 oxidase (*n* = 22 paired samples from 11 patients; *D*) expressed as the rotenone-inhibitable fraction of lucigenin-enhanced chemiluminescence and the gp91 tat-inhibitable fraction of 2OH-ethidium by HPLC, respectively, were increased in POST atrial samples. **P < 0.01 and ***P < 0.001 vs. PRE, by the Wilcoxon matched-pairs signed rank test (*C*) or the paired Student’s *t*-test (*D*). Data are shown as median and IQR (*A* and *C*) or mean and SD (*B* and *D*). RLU, relative light units.

**Figure 2:**
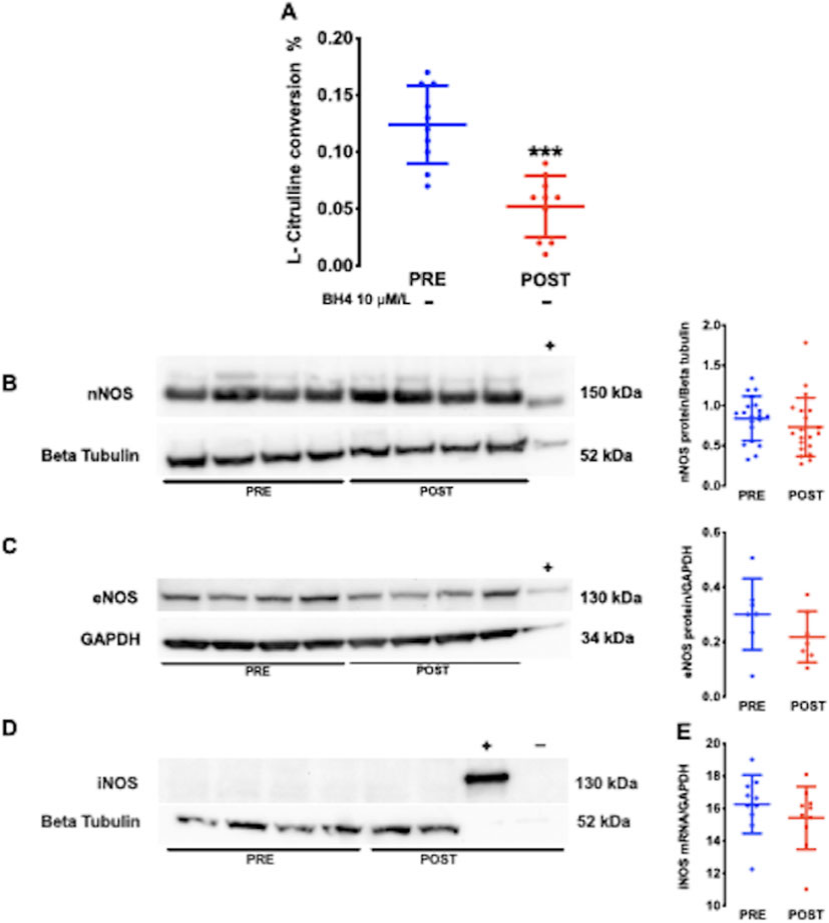
Atrial NOS activity significantly decreased in POST samples (*n* = 20 paired samples from 10 patients; *A*). ***P < 0.001 vs. PRE, by paired Student’s *t*-test. Atrial nNOS and eNOS protein content was unaltered after CPB and reperfusion (*B* and *C*) while iNOS protein was absent (*D*) and mRNA below the detection threshold (*E, n* = 14–40 samples from 7 to 20 patients). There were no significant differences between PRE and POST (*B*and *C*) by paired Student’s *t*-test. Data are shown as mean and SD (*A*–*D*). nNOS positive control (+) was murine brain tissue; iNOS positive control (+) were primary murine macrophages treated with LPS; Negative control (−) were primary murine macrophage in the absence of LPS stimulation. eNOS positive control (+) was human saphenous vein homogenate.

Atrial superoxide production, as measured by both lucigenin-enhanced chemiluminescence and 2-OH ethidium detection by HPLC, was significantly increased after CPB and reperfusion (*Figure 1A and B* and Supplementary material online, *Figure S3*). The fraction of atrial superoxide inhibited by the mitochondrial complex I inhibitor, rotenone, or the specific NOX2 inhibitor, gp91-ds-tat-peptide, was significantly higher in POST samples (*Figure 1C and D* and Supplementary material online, *Figure S4*), suggesting that both mitochondrial and NOX2 oxidases contribute to the rise in atrial superoxide production after reperfusion. By contrast, atrial NOS activity was significantly lower in POST samples (*Figure 2A*).

Atrial Rac-1 activity, NOX2 and NOX4 protein content (Supplementary material online, *Figure S5*) did not differ significantly after reperfusion nor did the endothelial and neuronal isoforms of NOS (nNOS and eNOS, respectively, *Figure 2B and C*). The uric acid-inhibitable fraction of luminol chemiluminescence was unaltered after reperfusion (Supplementary material online, *Figure S6*) and protein (*Figure 2D*) and mRNA (*Figure 2E*) of inducible NOS isoform (iNOS) were undetectable or below the detection threshold, respectively, in both PRE and POST right atrial samples.

### 3.2 Atrial GCH-1 activity and BH_4_ content are lower after CPB and reperfusion

**Figure 3:**
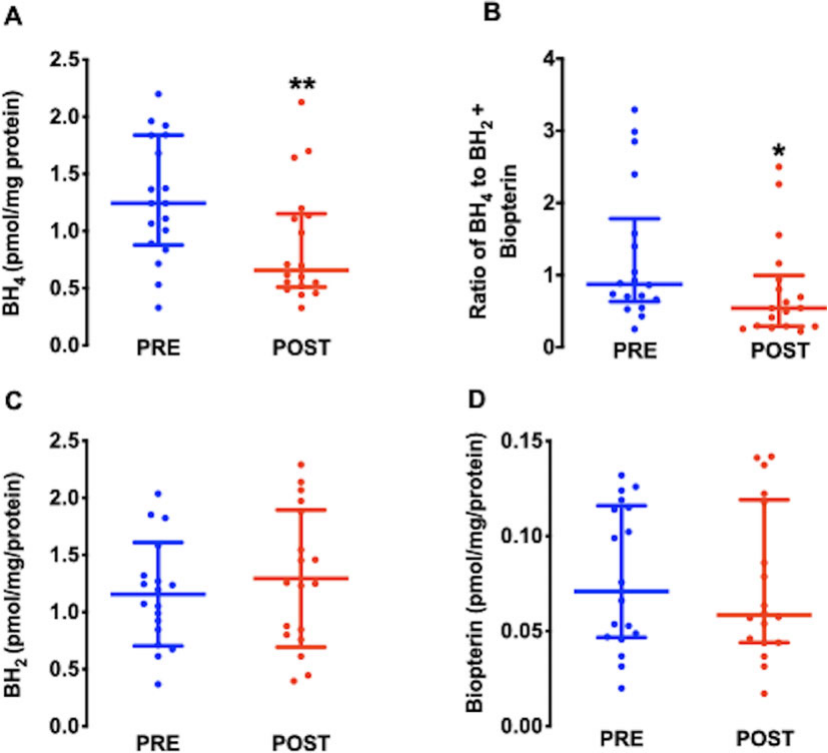
Atrial BH_4_ content decreased after CPB and reperfusion (*A*), whereas BH_2_ (*C*) and biopterin levels (*D*) were unaltered resulting in a reduction in the ratio of BH_4_ to BH_2_ + Biopterin (*B*), *n* = 36 paired samples from 18 patients. **P < 0.01, *P < 0.05 vs. PRE in *A* and *B* by Wilcoxon matched pairs sign rank test. *C* and *D* PRE-POST differences were not significant by paired Student’s t -test (*C*) or the Wilcoxon matched pairs sign rank test (*D*). Data are shown as median and IQR (*A–D*) or mean and SD (*C*).

**Figure 4:**
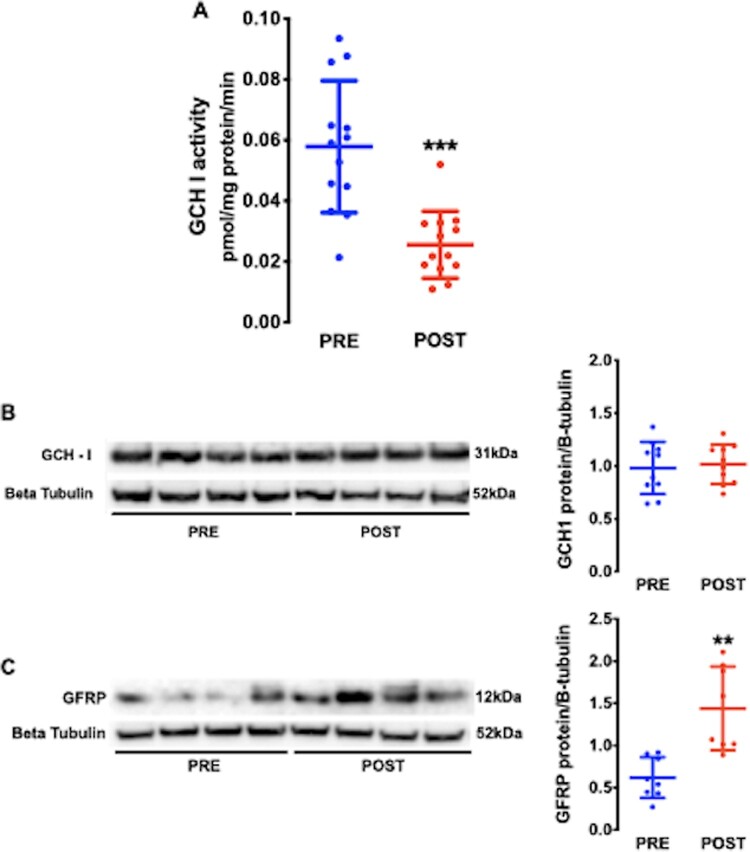
(*A*) Atrial GCH-1 activity decreased after CPB and reperfusion, *n* = 26 paired samples from 13 patients. ***P < 0.001 vs. PRE, by paired Student’s t test. (*B*) The atrial protein level of GCH-1 was unaltered whereas atrial GFRP (*C*) was significantly increased after CPB and reperfusion, *n* = 16—20 samples from 8 to 10 patients. **P < 0.01 vs. PRE by paired Student’s *t*-test. Data are shown as mean and SD.

Both BH_4_ content (*Figure 3A*) and the ratio of BH_4_ to its oxidized products, dihydrobiopterin or (BH_2_) and biopterin, were significantly lower (*Figure 3B*) in POST atrial samples. The BH_4_ oxidized products were unchanged (*Figure 3C* and *D*), suggesting a primary reduction in BH_4_ synthesis after CPB and reperfusion. In keeping with this hypothesis, the activity of the rate-limiting enzyme in the synthesis of BH_4_, GCH-1 was significantly reduced in POST atrial samples (*Figure 4A*) in the absence of changes in GCH-1 protein content (*Figure 4B*). By contrast, the atrial content of the GCH-1 feedback regulatory protein (GFRP), which mediates the inhibition of GCH-1 activity by BH_4_,^23^ was increased in POST samples (*Figure 4C*).

**Figure 5:**
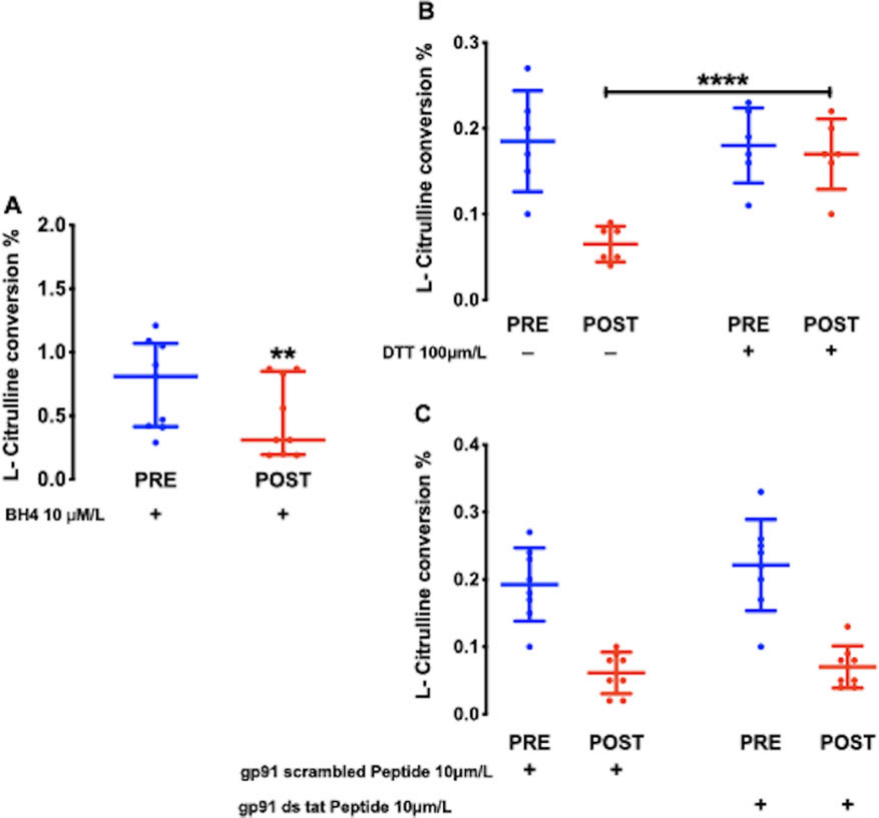
(*A*) The reduction in atrial NOS activity in POST samples (*n* = 18 paired samples from 9 patients) is not abolished by pre-treatment with BH_4_, **P < 0.01 vs. PRE by Wilcoxon matched pairs sign rank test. (*B*) Treatment with the reducing agent, dithiothreitol (DTT) restored NOS activity in POST samples, *n* = 12 paired samples from 6 patients; ****P < 0.0001, by analysis of covariance (ANCOVA) adjusted for PRE measurements, whereas (*C*) NOX2 inhibition with gp91ds tat vs. scrambled peptide did not, *n* = 16 paired samples from 8 patients by ANCOVA adjusted for PRE measurements. Data are expressed as median and IQR (*A*) or mean and SD (*B* and *C*).

To elucidate the role of BH_4_ deficiency in the reduction of NO synthesis in POST samples, we repeated measurements of NOS activity after incubation with BH_4_ (10 μmol/L). BH_4_ supplementation increased NOS activity overall but failed to abolish the difference between PRE and POST samples (*Figure 5A*). By contrast, pre-treatment with the reducing agent dithiothreitol (DTT) was effective in restoring NOS activity in POST samples without affecting the PRE measurement (*Figure 5B*). These findings suggest that the reduction in NOS activity following CPB and reperfusion may be mediated by a mechanism susceptible to reversal by DTT, i.e. a direct redox modification of the NOS protein. Although NOX2 contributed to the increase in superoxide production in POST samples (*Figure 1D*), NOX2 inhibition had no significant effect on PRE and POST atrial NOS activity (*Figure 5C*).

### 3.3 STARR: a randomized, double-blind placebo-controlled trial of perioperative atorvastatin

One hundred and ninety-nine patients scheduled for elective cardiac surgery on CPB were screened for inclusion in this study (Supplementary material online, *Figure S1B*), and 80 eligible patients were randomized to receive either atorvastatin (80 mg od) or placebo in a double-blind fashion. Following randomization, surgery was cancelled in 13 patients (15% assigned to atorvastatin and 18% assigned to placebo), and two patients withdrew consent. About 66% of randomized patients receiving surgery had a CABG (8% combined with AVR) and 34% AVR (Supplementary material online, *Table S1*).

The median pre - and post-operative duration of randomized treatment in both groups was 2 days (95% CI 1–5) and 5 days (95% CI 2–5), respectively. There were no differences in patient characteristics at randomization between treatment groups (Supplementary material online, *Table S1*). Peri- and post-operative management of patients in both groups was similar (Supplementary material online, *Table S2*), and there were no significant differences in in-hospital outcomes between groups (Supplementary material online, *Table S3*). Both TnI and NT-proBNP increased at 72 h after the surgery, with no significant differences between groups (*Table 1*). Patients allocated to atorvastatin had a greater reduction in LDL cholesterol at 72 h after surgery than those in the placebo arm [adjusted mean difference of 0.47 mM/L 95% CI (0.19–0.75), *P* = 0.001; *Table 1*].

**Table 1:** STARR - secondary endpoints

	Placebo	Atorvastatin	Adjusted mean difference (95% CI)	*P* value
Patients (*n*)	24	24		
LDL Cholesterol (mM/L)				
Baseline	2.18 (0.96)	2.10 (0.80)		
At 72 h	1.47 (0.62)	0.96 (0.62)	0.47 (0.19–0.75)	0.001
NT-proBNP (pg/mL)				
Baseline	2265 (2361)	2902 (4439)		
At 72 h	10 867 (6202)	13 158 (8280)	-1854.41 (−5926.75 to 2217.93)	0.36
Troponin I (ng/mL)				
Baseline	0.31 (0.61)	0.30 (0.50)		
At 72 h	0.75 (1.92)	0.42 (0.64)	0.32 (−0.33 to 0.96)	0.33
Creatinine (mM/L)				
Baseline	77.92 (18.05)	81.54 (18.11)		
At 72 h	88.79 (35.73)	103.20 (63.63)	-0.59 (−38.97 to 17.79)	0.46

Values are mean and standard deviation. *P* values derived by ANCOVA with adjustment for baseline values.

### 3.4 Perioperative atorvastatin prevents post-reperfusion changes in atrial superoxide generation and NOS activity but has no effect on post-operative AERP

**Figure 6:**
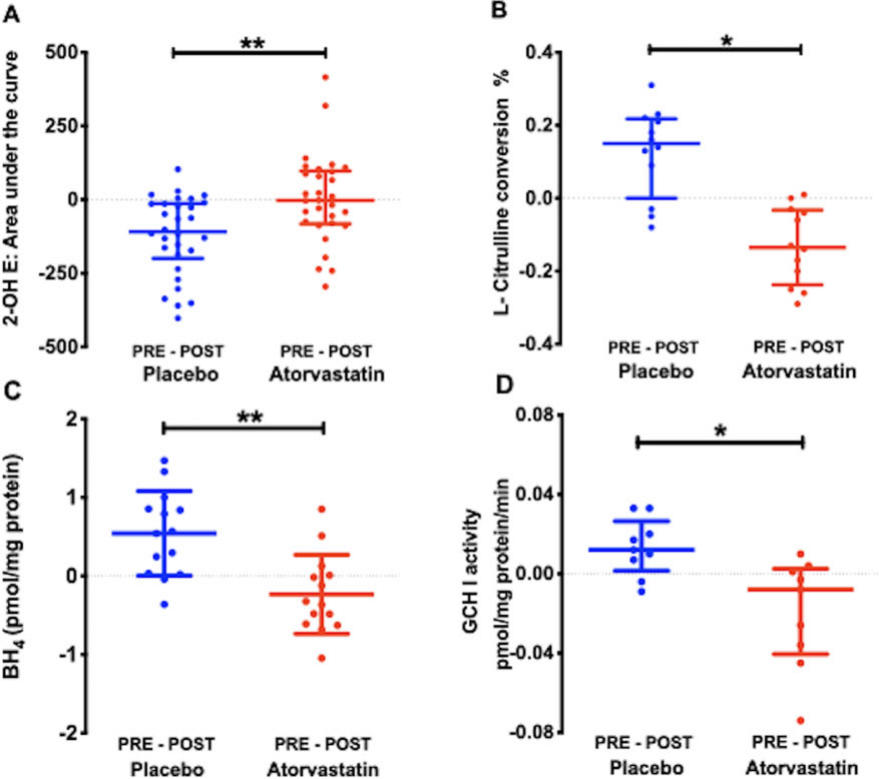
Differences between right atrial superoxide production, NOS activity, BH_4_ content and GCH-1 activity in PRE and POST samples from patients allocated to atorvastatin (80 mg od) or placebo. Treatment with atorvastatin prevented the increase in superoxide production (*A*) in POST atrial samples vs. placebo (*n* = 60 paired samples from 30 patients in each group; **P < 0.01, by ANCOVA) and the reduction in NOS activity (*B, n* = 24 paired samples from 12 patients; *P < 0.05 vs. placebo, by ANCOVA). Atorvastatin prevented the reduction in atrial BH_4_ content (*n* = 28 paired samples from 14 patients; *C*) and GCH-1 activity (*n* = 18 paired samples from 9 patients; *D*) in POST samples vs. placebo. **P < 0.01, *P < 0.05 vs. placebo, by ANCOVA. Data are shown as median and IQR (*A, B,* and *D*) or mean and SD (*C*).

Atorvastatin therapy prevented the increase in atrial superoxide production and reduction in NOS activity observed in the POST atrial samples of patients allocated to placebo (*Figure 6A and B* and Supplementary material online, *Figure S7*). In atrial samples from both treatment groups, atorvastatin prevented the reduction in atrial BH_4_ content and GCH-1 activity in POST samples (*Figure 6C* and *D*) in the absence of differences in BH_2_, biopterin or the ratio of BH_4_ to its oxidised products (Supplementary material online, *Figure S8*).

**Figure 7:**
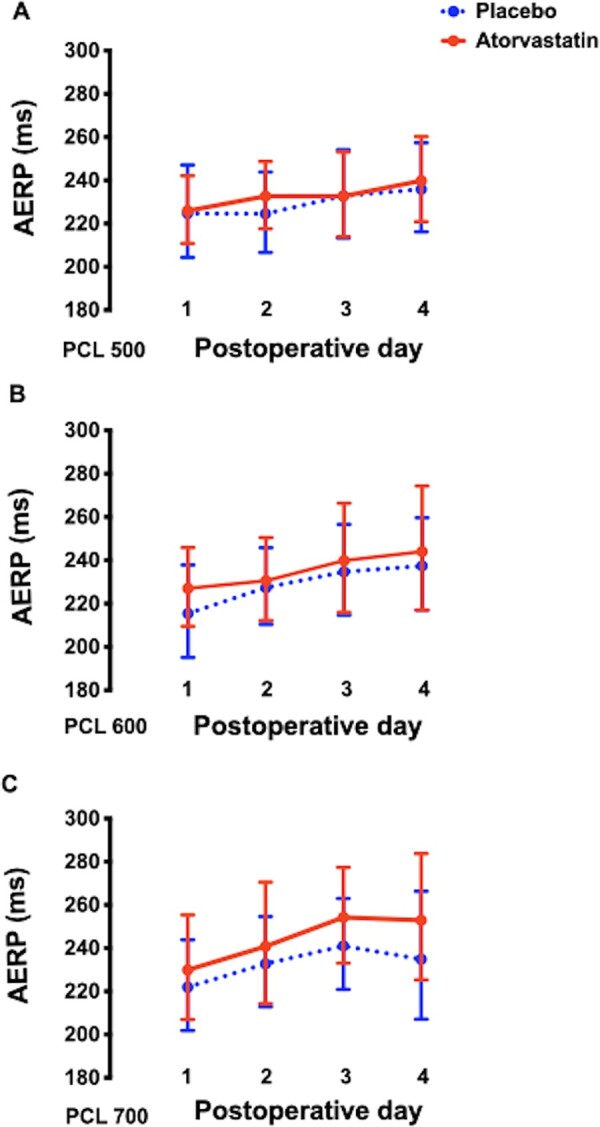
Average right atrial effective refractory period on first four postoperative days at three pacing cycle lengths. (*A*) pacing cycle length 500 ms, 14 patients allocated to atorvastatin and 15 patients allocated to placebo; (*B*) pacing cycle length 600 ms, 14 patients allocated to atorvastatin and 15 patients allocated to placebo; (*C*) pacing cycle length 700 ms, 10 patients allocated to atorvastatin, and 12 patients allocated to placebo; *P*  = 0.83, *P*  = 0.87 and *P*  = 0.75 by mixed model linear regression analysis after log transformation. Back transformed data are shown as geometric mean ± 95% confidence interval. AERP, atrial effective refractory period; PCL, pacing cycle length.

To evaluate whether preserving the atrial nitroso-redox balance in POST samples in the atorvastatin treated patients had an impact on atrial refractoriness, the right AERP was measured serially over the first four postoperative days in 29 patients (Supplementary material online, *Table S4A* and *B*). As shown in *Figure 7*, the AERP tended to lengthen over the first four post-operative days but did not differ between the treatment groups. There was no significant difference between the slopes of the linear regression lines of best-fit representing post-operative changes in AERP in the two groups across the three pacing cycle lengths (Supplementary material online, *Table S4C*).

## 4. Discussion

The present study demonstrates that perioperative atorvastatin treatment prevents the perioperative myocardial nitroso-redox imbalance in patients undergoing on-pump cardiac surgery, as assessed in samples of the right atrial appendage obtained before cardioplegia, and after CPB and reperfusion. However, atorvastatin treatment has no impact on troponin release, NT-proBNP levels or post-operative right atrial refractoriness and, by implication, myocardial injury, LV function, and the development of a functional substrate favouring post-operative AF. Together with findings from a large randomized controlled trial indicating that perioperative rosuvastatin (20 mg od) does not reduce the incidence of post-operative AF or any other in-hospital complication,^16^ our data indicate that the relationship between myocardial nitroso-redox imbalance and post-operative clinical outcomes may not be causal.

### 4.1 Mechanisms responsible for atrial nitroso-redox imbalance after CPB and reperfusion

At difference with experimental studies of severe I/R,^24^ the right atrial uric acid-inhibitable luminol chemiluminescence, which predominantly (but not exclusively)^25^ reflects peroxynitrite release,^26^ did not differ after CPB and reperfusion, suggesting that myocardial nitrosative stress is unlikely to play an important role in postoperative cardiac complications in humans. By contrast, atrial NOX2 and mitochondrial superoxide production were significantly increased following reperfusion. Typically, this would be expected to result in BH_4_ oxidation and lead to a reduction in NOS activity^6^; however, POST atrial BH_4_ was reduced in the absence of an increase in its most abundant oxidized products; moreover, the decrease in NOS activity post-reperfusion was not reversed by supplementing BH_4_. By contrast, DTT restored atrial NOS activity in POST samples, suggesting that the atrial oxidative environment may lead to redox-sensitive modification of eNOS, such as S-glutathionylation, as previously reported in the vascular endothelium in the context of I/R injury.^27^^,^^28^

Our previous findings indicated that nNOS accounts for most of the NO synthesis in the atrial and ventricular myocardium^11^^,^^29^; it is, therefore, possible that redox-mediated post-translational modifications of nNOS may have contributed to the reduction in total atrial NOS activity after CPB and reperfusion; however, S-glutathionylation of the nNOS reductase domain has yet to be demonstrated. Other mechanisms, such as altered NOS phosphorylation^30^ or reduced bioavailability of L-arginine,^31^ are less likely to have contributed significantly to a reduction NOS activity since treatment with DTT was sufficient to recover NOS activity in POST samples by more than 95%.

NOX2 and mitochondrial oxidases contributed significantly to the overall increase in atrial superoxide production after CPB and reperfusion. It has been reported that NOX2-derived superoxide may stimulate the mitochondrial ROS production^32^; the latter, in turn, may enhance NOX activity following hypoxia.^32^^,^^33^ NOX2 inhibition, however, did not restore NOS activity following CPB and reperfusion, suggesting that preservation of NOS activity in patients receiving atorvastatin may be underpinned by a different mechanism or that, once developed, NOS oxidative modifications do not reverse following NOX2 inhibition.

Under physiological conditions, GCH -1 expression and activity determine the rate of BH_4_ synthesis.^23^ When GCH-1 is complexed with GFRP, BH_4_ binds to a selective pocket at the interface of the protein complex and induces allosteric changes resulting in negative feedback regulation of its synthesis.^34^ Over-expression of recombinant human GFRP in a murine endothelial cell line had no effect on BH_4_ or NO levels under basal conditions but attenuated the increase in BH_4_ and NO synthesis in response to lipopolysaccharide and cytokines, suggesting GFRP may regulate the BH_4_ synthesis and NOS activity in the presence of inflammation.^35^ Lending support to this notion, incubating human endothelial cells with hydrogen peroxide increased GFRP mRNA and reduced BH_4_ levels.^36^ In our study, the increase in GFRP protein abundance in POST atrial samples was associated with a reduction in BH_4_ levels and in GCH-1 and NOS activity. Supplementing a saturating concentration of BH_4_ (10 μmol/L)^37^ increased NOS activity overall but failed to abolish the reduction in NOS activity in POST samples. By contrast, infusion of liposomal BH_4_ before the onset of ischaemia in an *in vivo* myocardial I/R injury rat model has been reported to prevent eNOS dysfunction.^38^

### 4.2 Atorvastatin and myocardial nitroso-redox imbalance

In addition to their LDL-lowering effects, statins exert a rapid antioxidant effect that is, at least partly, mediated by NOX2 inhibition.^39^ Whether the pleiotropic effects of statins confer cardioprotection in patients undergoing on-pump cardiac surgery remains a matter of debate. We found that pre- and perioperative therapy with atorvastatin prevented the rise in atrial superoxide production and the reduction in atrial NOS activity and BH4 content after CPB and reperfusion. Other putative mechanisms by which statin therapy may preserve atrial nitroso-redox balance after CPB and reperfusion, include increasing eNOS protein content and its phosphorylation at the serine 1177 site whilst reducing p38 signalling, as reported in an open trial of simvastatin (20 mg od) in 151 patients undergoing non-coronary cardiac surgery.^40^ In STARR, preservation of the atrial nitroso-redox balance by statins was not associated with a reduction in perioperative troponin-I, NT-Pro BNP, or in any other post-operative complications. Although our study was not powered to detect a moderate difference in post-operative outcomes, the results are in keeping with those of a much larger double-blind placebo-controlled randomized trial which showed that perioperative rosuvastatin (20 mg od) had no effects on postoperative AF, perioperative myocardial injury, or LV function.^16^

### 4.3 Atorvastatin and postoperative atrial refractoriness

AERP is a measure of atrial refractoriness; a short AERP is expected to promote functional re-entry and contribute to the atrial electrical substrate that promotes AF. Experimental evidence supporting a direct role of reactive oxygen species in altering atrial electrical characteristics is scanty. Both ascorbate (an antioxidant and peroxynitrite decomposition catalyst) and high-dose simvastatin have been reported to prevent AERP shortening induced by atrial tachypacing in dogs.^15^^,^^41^ In a canine model of sterile pericarditis, perioperative administration of atorvastatin was associated with a longer postoperative AERP.^42^ In STARR, prevention of the atrial NO-redox imbalance by atorvastatin after CPB and reperfusion did not alter the AERP after surgery, suggesting that the atrial NO-redox status is unlikely to be causally linked to the atrial electrical properties harbouring AF in patients after cardiac surgery.

### 4.4 Limitations

The small amount of PRE and POST atrial tissue available for our studies limited the number of experiments we could undertake to elucidate the mechanisms underlying the atrial nitroso-redox imbalance after CPB and reperfusion and its prevention by perioperative atorvastatin treatment. In particular, it precluded the investigation of the putative role of other oxidase systems in NOS dysfunction as well as the study of redox-mediated post-translational modifications of NOS enzymes. For instance, S-glutathionylation of critical cysteines of the eNOS reductase domain can lead to uncoupled NOS activity in response to hypoxia-reperfusion, resulting in reduced NO synthesis and increase superoxide production.^28^

We did not measure plasma markers of lipid or protein oxidation in the current study, as we have previously shown that they do not predict the new onset of AF after cardiac surgery nor are they correlated with myocardial ROS production.^1^^,^^8^ Serum levels of nitrotyrosine (reflecting the activity of reactive nitrogen species) have been reported to be lower in patients with coronary artery disease on statin therapy^43^; however, whether this finding has a bearing on the beneficial effect of statins remains to be ascertained.

Depending on the underlying electrophysiological substrate, different mechanisms can contribute to the pathogenesis of postoperative AF.^2^ For instance, a greater dispersion of atrial refractoriness and atrial conduction delay may predispose to postoperative AF^44^; unfortunately, these parameters could not be collected in our patients. Similarly, as routine surgical practice only involved insertion of right atrial and ventricular pacing leads, we were unable to measure left AERP.

## 5. Conclusions

Short-term statin therapy, at the doses conventionally used to lower LDL-cholesterol, preserves atrial NO-redox balance in patients who had cardiac surgery on CPB. However, these findings had no significant impact on troponin release or atrial refractoriness, casting some doubts on the pathogenic role of myocardial nitroso-redox imbalance on postoperative AF and myocardial injury in patients undergoing on-pump cardiac surgery. Overall, our findings are consistent with randomized evidence indicating that perioperative statin treatment does not reduce the incidence of post-operative AF.^16^^,^^22^

## Supplementary material

Supplementary material is available at *Cardiovascular Research* online.

## Author’s contributions

R.J.: Patient recruitment, biobanking, atrial effective refractory period measurements, superoxide measurements, immunoblotting, high performance liquid chromatography, analysis of the data, drafted first version of the manuscript. M.J., Y.B., and T.B.: Atrial effective refractory period measurements. S.R., N.G., J.S., K.N., and R.C.: Immunoblotting and quantitative polymerase chain reaction. N.P.: Echocardiography measurements. M.C. and K.C.: High performance liquid chromatography. A.R.: Statistical analysis. R.D., C.R., M.P., and R.S.: Biobanking. M.H.: Biomarker assays. B.C.: Had the original idea, developed the protocol in collaboration with clinical trials services unit (CTSU), University of Oxford, revised the manuscript and supervised R.J.

## Supplementary Material

cvaa302_Supplementary_Data

## Notes

Translational perspective Increased atrial ROS production is associated with both incident and prevalent AF, with experimental findings suggesting it may have a causal role in AF induction and AF-induced electrical remodelling. Statin therapy causes a reduction in myocardial and vascular ROS production and as such it may prevent the new onset of AF after cardiac surgery. In patients undergoing on-pump cardiac surgery, we show that perioperative administration of statins prevents myocardial nitroso-redox imbalance after reperfusion without affecting atrial refractoriness or perioperative myocardial injury. These findings suggest that targeting myocardial nitroso-redox imbalance would be unlikely to prevent postoperative complications in patients undergoing on-pump cardiac surgery.
