# Supplementary material for: Atrial nitroso-redox balance and refractoriness following on-pump cardiac surgery: a randomized trial of atorvastatin
Source: Cardiovasc Res. 2020 Oct 24;118(1):184–95. doi: 10.1093/cvr/cvaa302 (PMC8752359; doi:10.1093/cvr/cvaa302)
Supplement: cvaa302_Supplementary_Data [file cvaa302_supplementary_data.pdf]

SUPPLEMENTARY MATERIAL

Atrial nitroso-redox balance and refractoriness following on-pump cardiac surgery: A randomised trial of atorvastatin

Jayaram, NO-redox balance and on-pump cardiac surgery

Raja Jayaram et al

CONSORT Checklist

Figure 1A

CONSORT 2010 checklist of information to include when reporting a randomised trial*

Section/Topic	Item No	Checklist item	Reported on page No
**Title and abstract**			
	1a	Identification as a randomised trial in the title	1
	1b	Structured summary of trial design, methods, results, and conclusions (for specific guidance see CONSORT for abstracts)	2
**Introduction**			
Background and objectives	2a	Scientific background and explanation of rationale	4
	2b	Specific objectives or hypotheses	4
**Methods**			
Trial design	3a	Description of trial design (such as parallel, factorial) including allocation ratio	5
	3b	Important changes to methods after trial commencement (such as eligibility criteria), with reasons	
Participants	4a	Eligibility criteria for participants	5
	4b	Settings and locations where the data were collected	5
Interventions	5	The interventions for each group with sufficient details to allow replication, including how and when they were actually administered	5
Outcomes	6a	Completely defined pre-specified primary and secondary outcome measures, including how and when they were assessed	5
	6b	Any changes to trial outcomes after the trial commenced, with reasons	
Sample size	7a	How sample size was determined	7
	7b	When applicable, explanation of any interim analyses and stopping guidelines	
Randomisation:			
Sequence generation	8a	Method used to generate the random allocation sequence	5
	8b	Type of randomisation; details of any restriction (such as blocking and block size)	
Allocation concealment mechanism	9	Mechanism used to implement the random allocation sequence (such as sequentially numbered containers), describing any steps taken to conceal the sequence until interventions were assigned	5
Implementation	10	Who generated the random allocation sequence, who enrolled participants, and who assigned participants to interventions	5
Blinding	11a	If done, who was blinded after assignment to interventions (for example, participants, care providers, those assessing outcomes) and how	5
	11b	If relevant, description of the similarity of interventions	
Statistical methods	12a	Statistical methods used to compare groups for primary and secondary outcomes	7-8
	12b	Methods for additional analyses, such as subgroup analyses and adjusted analyses	7-8
**Results**			
Participant flow (a diagram is strongly recommended)	13a	For each group, the numbers of participants who were randomly assigned, received intended treatment, and were analysed for the primary outcome	Figure1b, supplement
	13b	For each group, losses and exclusions after randomisation, together with reasons	Figure1b, supplement
Recruitment	14a	Dates defining the periods of recruitment and follow-up	
	14b	Why the trial ended or was stopped	
Baseline data	15	A table showing baseline demographic and clinical characteristics for each group	Table 1, supplement
Numbers analysed	16	For each group, number of participants (denominator) included in each analysis and whether the analysis was by original assigned groups	Figure 1b, supplement
Outcomes and estimation	17a	For each primary and secondary outcome, results for each group, and the estimated effect size and its precision (such as 95% confidence interval)	9-10
	17b	For binary outcomes, presentation of both absolute and relative effect sizes is recommended	9-10
Ancillary analyses	18	Results of any other analyses performed, including subgroup analyses and adjusted analyses, distinguishing pre-specified from exploratory	
Harms	19	All important harms or unintended effects in each group (for specific guidance see CONSORT for harms)	
**Discussion**			
Limitations	20	Trial limitations, addressing sources of potential bias, imprecision, and, if relevant, multiplicity of analyses	14
Generalisability	21	Generalisability (external validity, applicability) of the trial findings	
Interpretation	22	Interpretation consistent with results, balancing benefits and harms, and considering other relevant evidence	11-15
**Other information**			
Registration	23	Registration number and name of trial registry	2
Protocol	24	Where the full trial protocol can be accessed, if available	
Funding	25	Sources of funding and other support (such as supply of drugs), role of funders	5. 15

Figure 1B **CONSORT diagram**

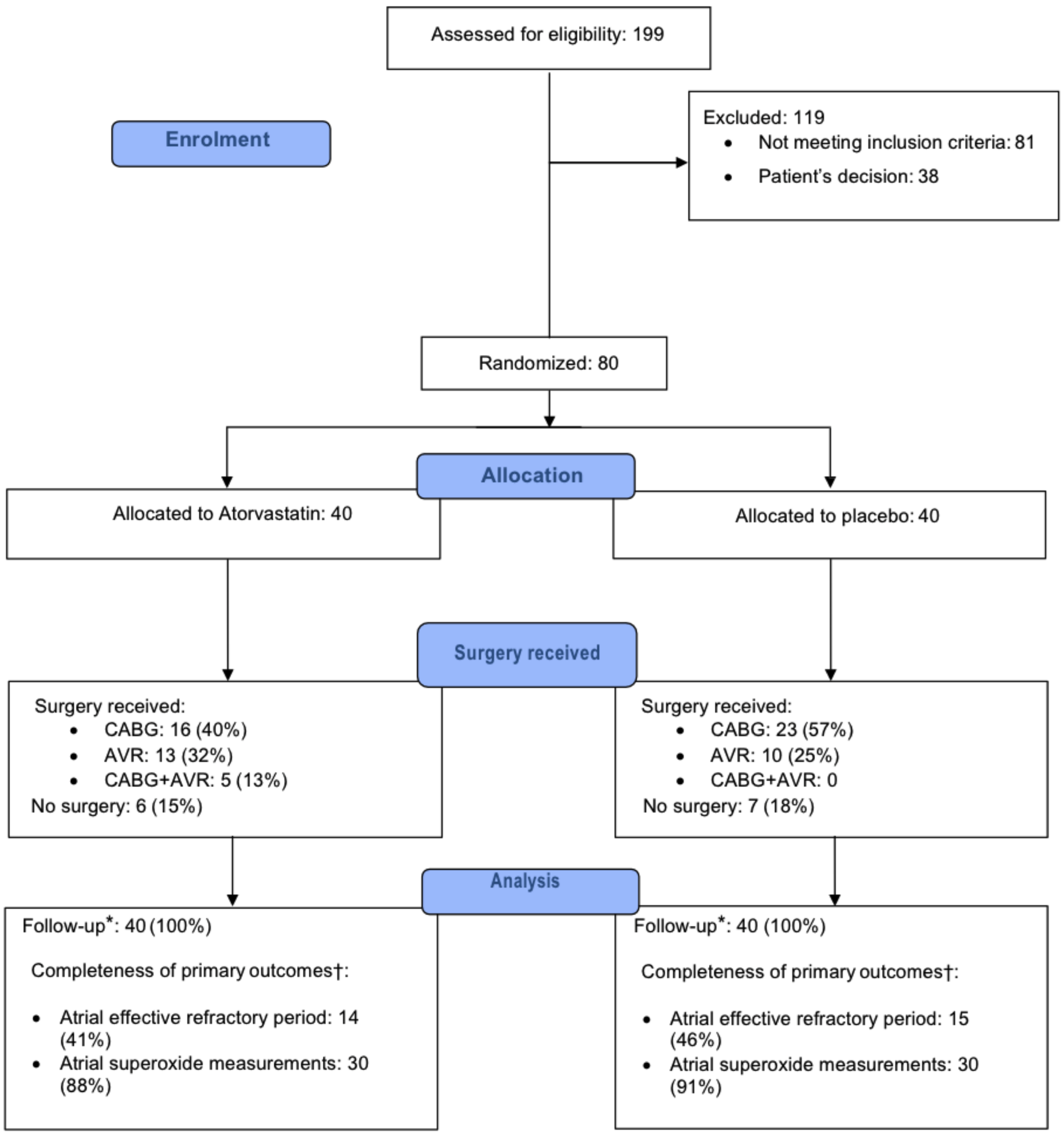

* Two patients withdrew consent
† In patients in whom surgery was undertaken

Figure. 2

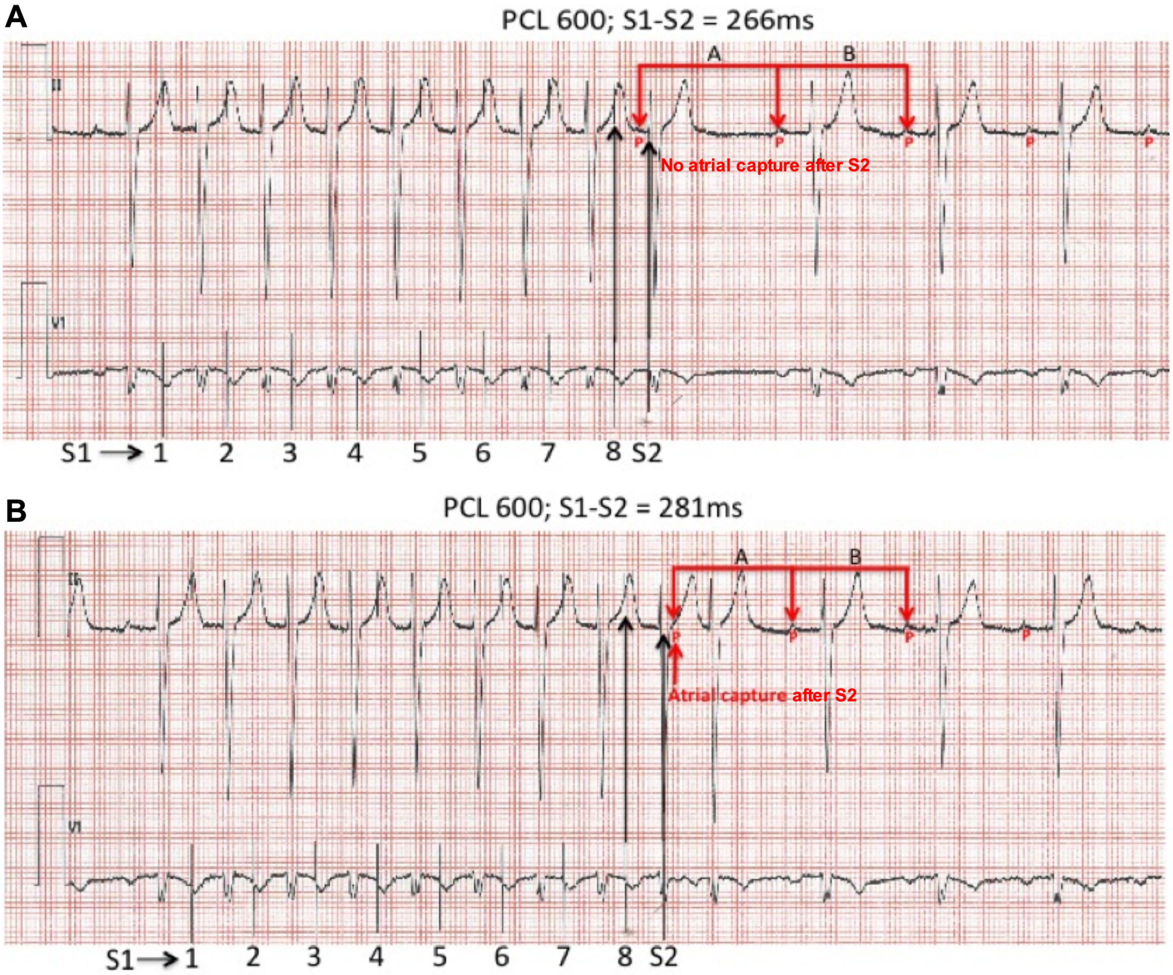
A
PCL 600; S1-S2 = 266ms
No atrial capture after S2
S1 → 1 2 3 4 5 6 7 8 S2
B
PCL 600; S1-S2 = 281ms
Atrial capture after S2
S1 → 1 2 3 4 5 6 7 8 S2

Figure 3

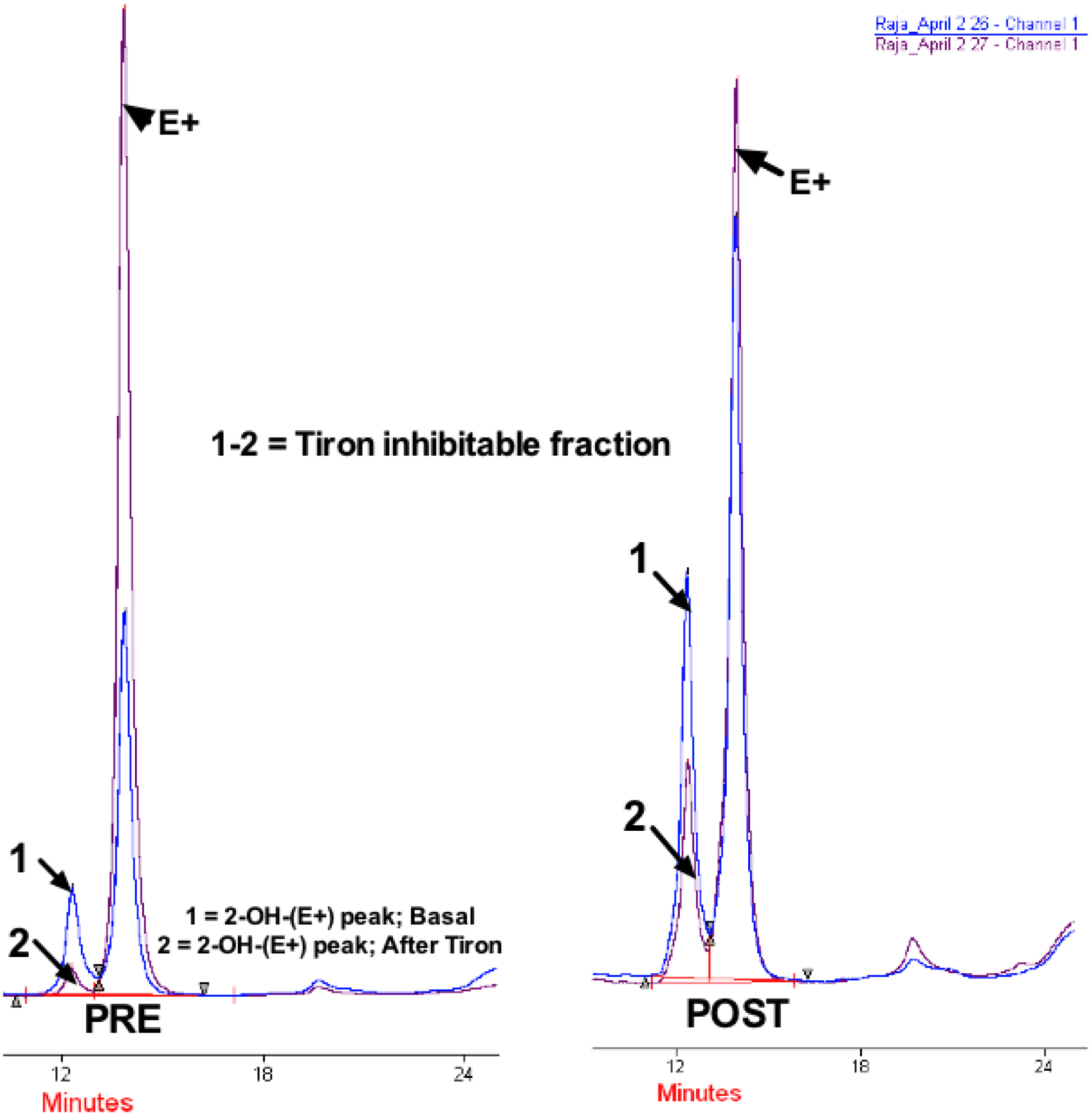

Raja_April 2 26 - Channel 1
Raja_April 2 27 - Channel 1
E+
E+
1-2 = Tiron inhibitable fraction
1
2
1
2
1 = 2-OH-(E+) peak; Basal
2 = 2-OH-(E+) peak; After Tiron
PRE
POST
12
18
24
Minutes
12
18
24
Minutes

Figure 4

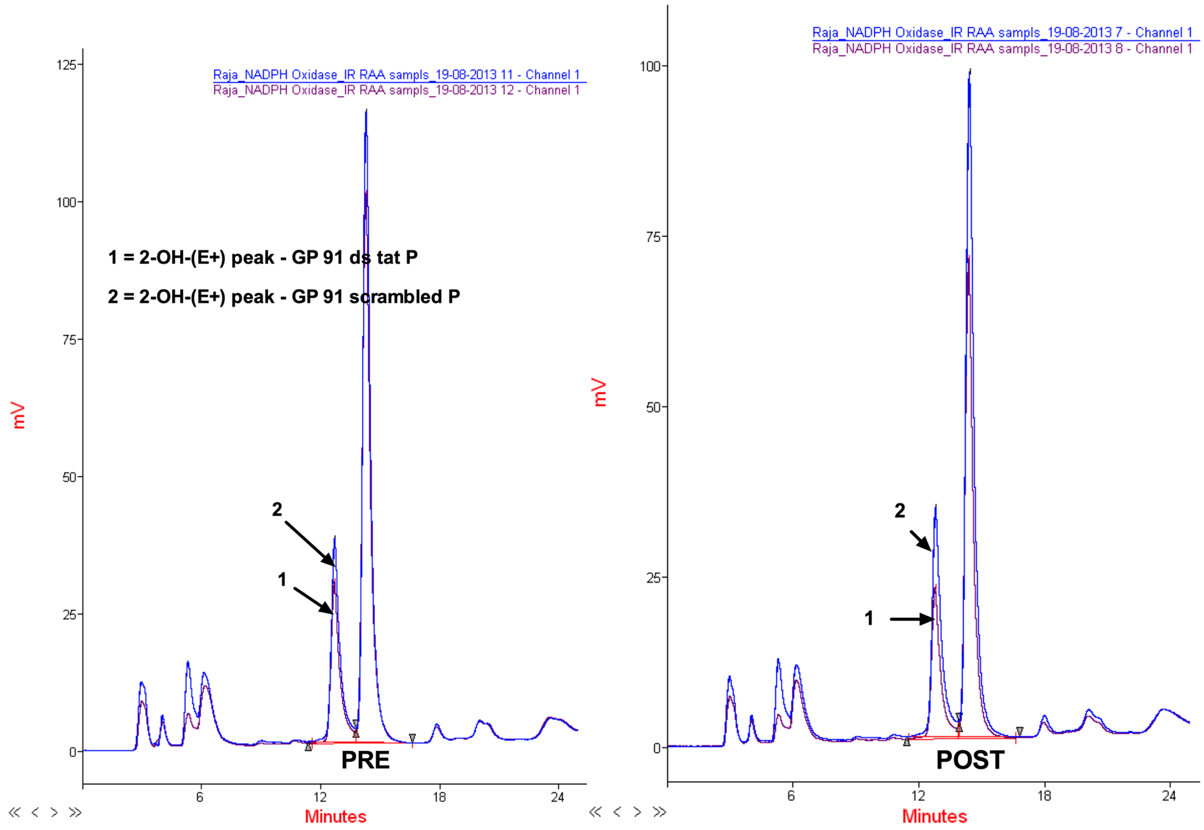

Raja_NADPH Oxidase_IR RAA sampls_19-08-2013 11 - Channel 1
Raja_NADPH Oxidase_IR RAA sampls_19-08-2013 12 - Channel 1
1 = 2-OH-(E+) peak - GP 91 ds tat P
2 = 2-OH-(E+) peak - GP 91 scrambled P
mV
Minutes
PRE
Raja_NADPH Oxidase_IR RAA sampls_19-08-2013 7 - Channel 1
Raja_NADPH Oxidase_IR RAA sampls_19-08-2013 8 - Channel 1
POST

Figure. 5

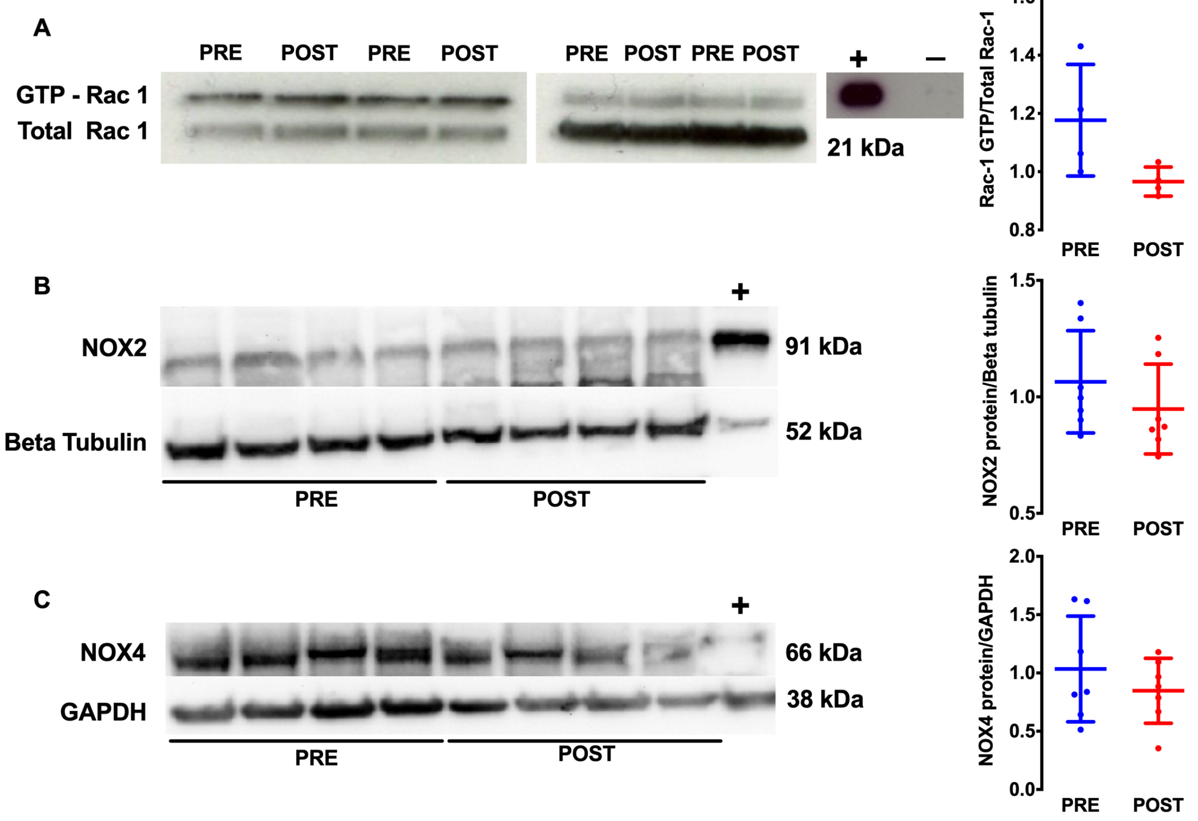
A
PRE
POST
PRE
POST
PRE
POST
PRE
POST
+
–
GTP - Rac 1
Total Rac 1
21 kDa
Rac-1 GTP/Total Rac-1
1.6
1.4
1.2
1.0
0.8
PRE
POST
B
+
NOX2
Beta Tubulin
91 kDa
52 kDa
PRE
POST
NOX2 protein/Beta tubulin
1.5
1.0
0.5
PRE
POST
C
+
NOX4
GAPDH
66 kDa
38 kDa
PRE
POST
NOX4 protein/GAPDH
2.0
1.5
1.0
0.5
0.0
PRE
POST

Figure. 6

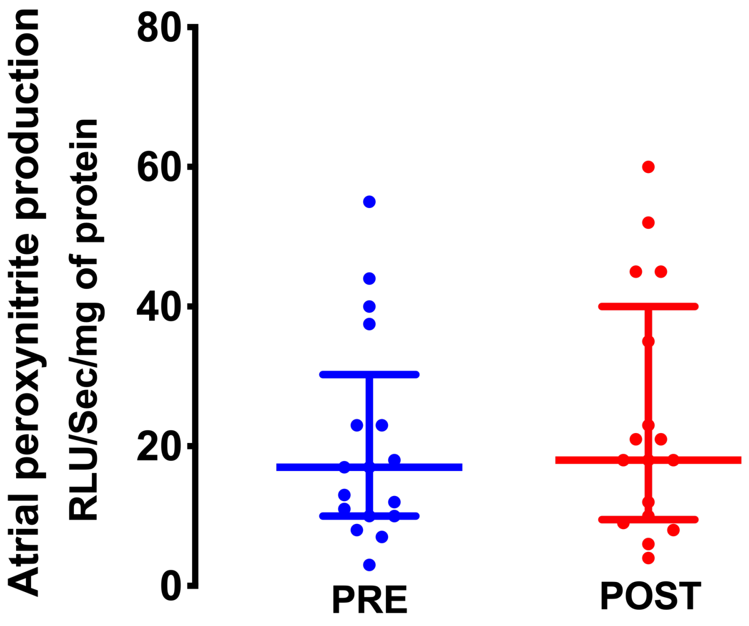
Atrial peroxynitrite production
RLU/Sec/mg of protein
80
60
40
20
0
PRE
POST

Figure 7

A

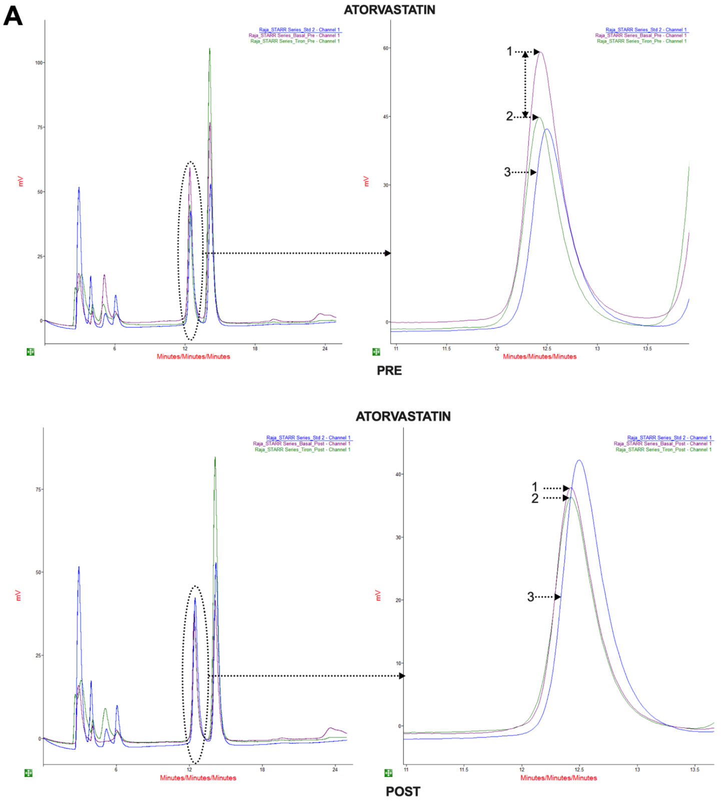

1 = 2-OH (E+) Peak; Basal

2 = 2-OH (E+) Peak; After Tiron 100mmol/L

3 = 2-hydroxyethidium [(2-OH (E+)] peak; standard

1 - 2 = Tiron inhibitable fraction

B

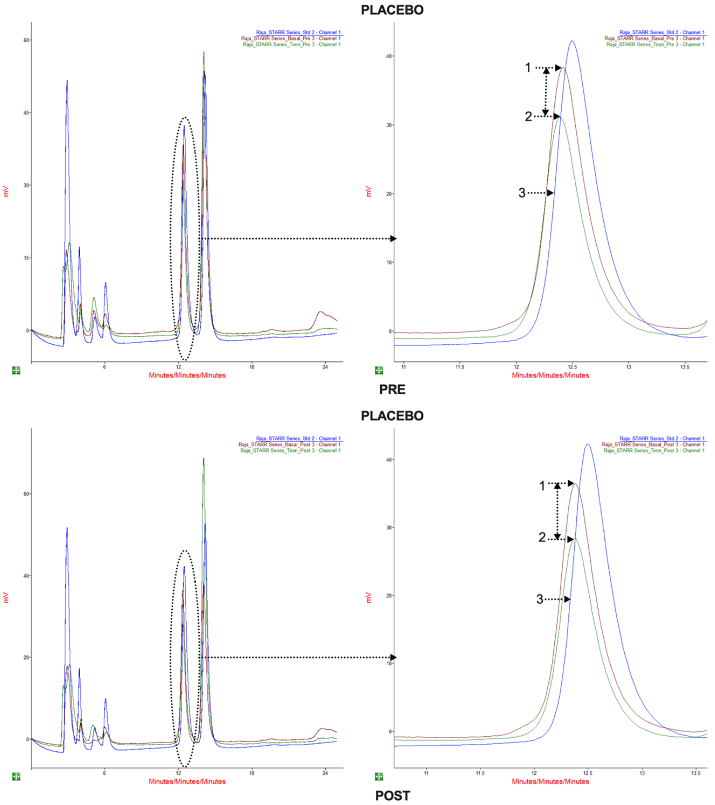

1 = 2-OH (E+) Peak; Basal

2 = 2-OH (E+) Peak; After Tiron 100mmol/L

3 = 2-hydroxyethidium [(2-OH (E+)] peak; standard

1 - 2 = Tiron inhibitable fraction

Figure. 8

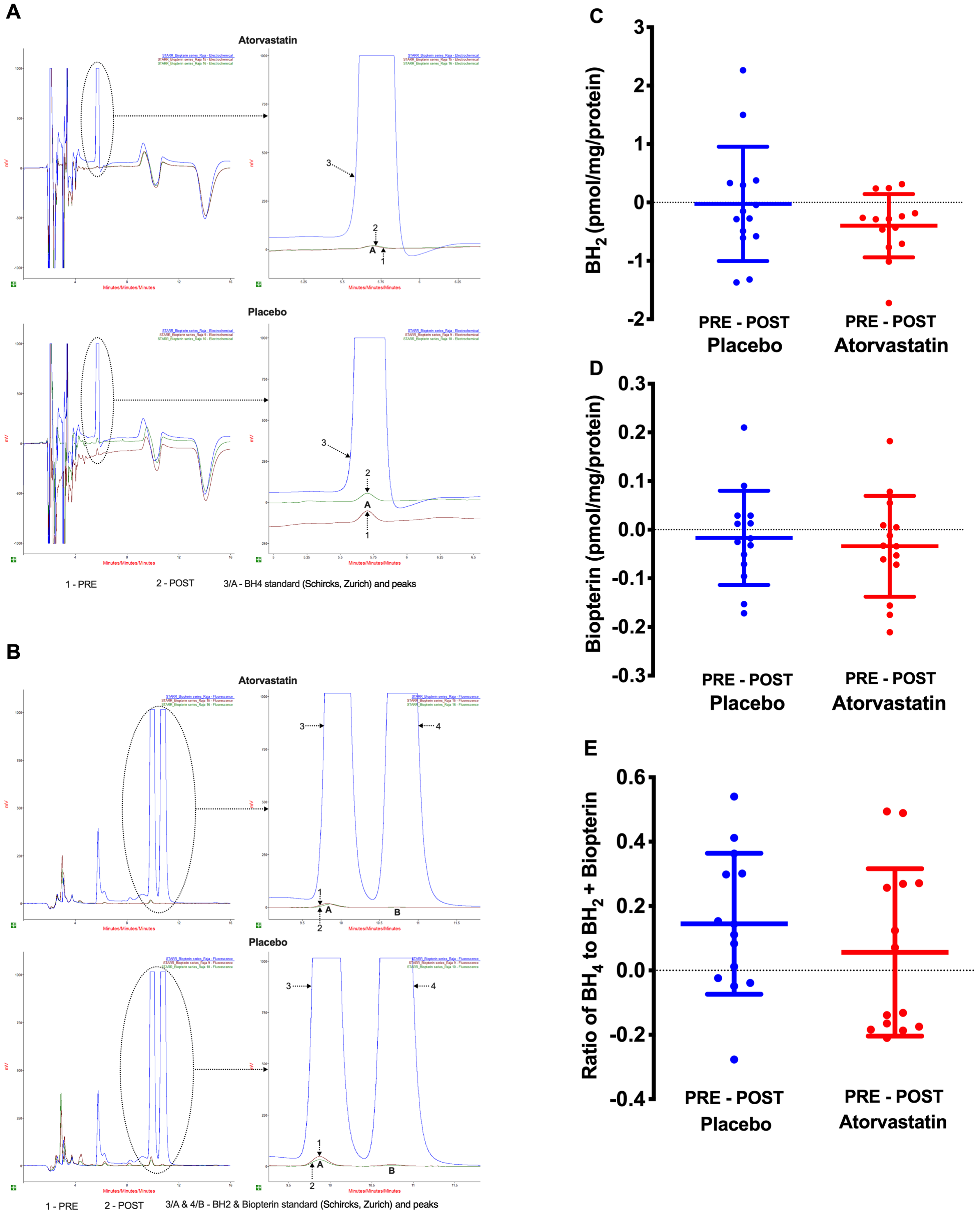
A
Atorvastatin
Placebo
Minutes/Minutes/Minutes
1 - PRE
2 - POST
3/A - BH4 standard (Schircks, Zurich) and peaks
B
Atorvastatin
Placebo
1 - PRE
2 - POST
3/A & 4/B - BH2 & Biopterin standard (Schircks, Zurich) and peaks
C
BH2 (pmol/mg/protein)
PRE - POST
Placebo
PRE - POST
Atorvastatin
D
Biopterin (pmol/mg/protein)
PRE - POST
Placebo
PRE - POST
Atorvastatin
E
Ratio of BH4 to BH2 + Biopterin
PRE - POST
Placebo
PRE - POST
Atorvastatin

Supplementary Figure 1A and B. Consort checklist and Diagram.

Supplementary Figure 2. A. Representative ECG recording obtained during programmed stimulation at a 600 ms pacing cycle length (PCL) with S1- S2 coupling interval of 266 ms showing failure of atrial capture following S2. The long black arrow indicates the point where an atrial extra stimulus (S2) introduced after every eighth conditioning train of 100 stimuli (S1) delivered at 2 x diastolic threshold. Red arrows indicate the presence of clearly visible 'p' wave on the continuous ECG.

B. A representative ECG recording obtained during programmed stimulation at 600ms PCL with S1-S2 coupling interval of 281 ms showing atrial capture evidenced by the presence of clearly visible 'p' wave on the continuous ECG and the sinus cycle length of the first return intrinsic beat after S2.

Supplementary Figure 3.

Representative HPLC chromatograms of 2- hydroxyethidium [2-OH-(E+)] in homogenates of right atrial samples obtained before (PRE) and after CPB and reperfusion (POST). 1 and 2 are 2- hydroxyethidium raw data traces obtained before and after treatment with Tiron (100mM/L) whilst 3 represents the ethidium peak (E+). The difference between 1 and 2, i.e., the Tiron-inhibitable fraction, was taken as a measurement of superoxide release in the respective samples.

Supplementary Figure 4

Representative HPLC chromatograms illustrating the gp91-ds-tat peptide-inhibitable fraction of superoxide release in homogenates of right atrial samples taken before (PRE) and after CPB (POST) and reperfusion. 1 and 2 are the peaks representing 2-

hydroxyethidium in the presence of the gp91-ds-tat peptide (10 μm/L) or a scrambled peptide (10 μm/L), respectively. The difference between the area under the peak of samples incubated with the scrambled peptide or with the gp91-ds-tat peptide (i.e., the gp91-ds-tat inhibitable fraction) was taken as a measurement of superoxide released by NOX2 NADPH oxidases.

Supplementary Figure 5

Atrial protein content of total and GTP-bound Rac-1 (**A**), NOX2 **(B),** and NOX4 **(C)** were unaltered after CPB and reperfusion (n = 8 - 20 samples from 4-10 patients; paired Student's t –test). For activated Rac-1, the positive control was GTPγS, i.e., a non-hydrolysable analog of GTP and the negative control (-) was GDP. Left ventricular tissue from mice with myocardial-specific NOX2 expression and murine kidney homogenates were used as positive controls (+) for NOX2 and NOX4, respectively. The data are shown as mean and SD. GTP - Guanosine triphosphate; GDP - Guanosine di phosphate.

Supplementary Figure 6 Uric acid-inhibitable fraction of luminol chemiluminescence (mostly reflecting peroxynitrite formation) in right atrial samples homogenates obtained before (PRE) and after (POST) CPB and reperfusion (n=38 paired samples from 19 patients). $P > 0.05$, by Wilcoxon matched-pairs signed rank test. The data are expressed as median and IQR.

Supplementary Figure 7.

Representative chromatograms of 2- hydroxyethidium in homogenates of right atrial samples obtained before (PRE) and after CPB and reperfusion (POST) from patients

allocated to **(A)** atorvastatin (80 mg od) or **(B)** placebo. In the enlarged panels on the right 1 and 2 are the 2- hydroxyethidium raw data traces obtained before and after treatment with Tiron (100 mM/L) and 3 is the 2- hydroxyethidium standard. The Tiron-inhibitable fraction represents the superoxide release in the respective sample.

Supplementary Figure 8. Representative HPLC chromatograms of BH4 **(A)** and BH2 and biopterin **(B)** in right atrial samples obtained before (PRE) and after CPB and reperfusion (POST) in patients allocated to atorvastatin (80 mg od) or placebo. In the enlarged panel on the right 1 and 2 are measurements of BH4, BH2 or biopterin PRE and POST, respectively. In panel **(A),** the peak marked with 3 is the BH4 standard (A). In panel **(B)**, the peaks marked with 3 and 4 are the BH2 (A) and biopterin (B) standards, respectively. Individual and average differences in BH_2 (**C**), biopterin (**D**) and the BH_4 to BH_2 + biopterin ratio **(E**) between PRE and POST atrial samples from patients allocated to atorvastatin (80 mg od) or placebo (n = 28 paired samples from 14 patients in each group**)**. Data are shown as mean and SD and comparisons were made using ANCOVA adjusted for PRE measurements.

Supplementary Table 1 Baseline characteristics in Study 1 and in STARR by randomised treatment allocation

	Study 1	STARR	
**Demographic and clinical variables**		**Atorvastatin**	**Placebo**
Patients, n	116	40	40
Age, years mean(sd)	69 (9)	64 (10)	65 (9)
Female,n(%)	27 (33)	8 (20)	7 (18)
Current smoker, n(%)	15 (13)	7 (18)	6 (15)
BMI, mean (sd)	27 (4)	29 (5)	28 (5)
Logistic Euroscore, mean (sd)	4 (3)	3 (2)	3 (2)
Left ventricular ejection fraction, % mean(sd)	59 (4)	61 (10)	62 (11)
Left atrial volume index in mL/m2, mean(sd)	--	30 (11)	26 (9)
**Pre-operative medications, n (%)**			
Beta blockers	83 (72)	20 (50)	23 (58)
NSAIDs/Steroids	8 (7)	1 (3)	1 (3)
Insulin	16 (14)	0	0
Antiplatelets	88 (76)	20 (50)	24 (60)
Anticoagulants	6 (5)	0	1 (3)
Calcium channel blockers	43 (37)	8 (20)	14 (35)
ACEi/ARB	82 (71)	25 (63)	26 (65)
Nitrates	52 (45)	10 (25)	6 (15)
Potassium sparing diuretics	5 (4)	0	0
Loop/Thiazide Diuretics	28 (24)	7 (18)	8 (20)
Statins	92 (79)	30 (75)	33 (83)
**Surgery* n (%)**			
CABG or CABG+AVR	96(82)	21 (53)	23 (57)
AVR	20(18)	13 (32)	10 (25)

* Only 67 randomized patients had surgery within the treatment window of STARR.

ACEi, Angiotensin converting enzyme inhibitors; ARB, Angiotensin receptor blockers; AVR, Aortic valve replacement; BMI, Body mass index; CABG, Coronary artery bypass surgery; NSAID, Nonsteroidal anti-inflammatory drugs.

Supplementary Table 2 STARR – Perioperative management

	Atorvastatin	Placebo
Patients, n	34	33
**Intraoperative variables**		
Cardiopulmonary bypass time (min) mean(sd)	88 (25)	81 (24)
Aortic cross clamp time (min) mean(sd)	57 (22)	46 (13)
**Peri/Intraoperative management**		
Intra-operative defibrillation, n(%)	4 (12)	2 (6)
Use of internal/external pacemaker, n(%)	16 (49)	17 (53)
Use of intra-aortic balloon pump, n(%)	0	0
Use of vasopressors/inotropes, n(%)	8 (24)	7 (22)
Surgical re-exploration, n(%)	2 (6)	0
Renal replacement therapy/dialysis, n(%)	0	0
Duration of ventilatory support (hours) mean(sd)	6 (1)	5 (1)
Duration of ICU stay (days) mean(sd)	2 (1)	2
**Postoperative treatment, n (%)**		
Beta blockers	29 (88)	31 (97)
Antiplatelets	1 (3)	1 (3)
NSAIDs/Steroids	2 (6)	0
Potassium supplements	33 (100)	30 (93)
Blood/blood products	8 (24)	4 (13)
ACEi/ARB	30 (91)	31 (97)
Amiodarone	12 (36)	7 (22)
Digoxin	1 (3)	1 (3)
Diuretics (includes potassium sparing)	30 (91)	28 (88)
Calcium channel blockers	9 (27)	4 (13)

ICU - Intensive care unit.

Supplementary Table 3 STARR - Postoperative complications.

	**Atorvastatin**	**Placebo**
Patients, n	34	33
**Postoperative outcomes***, **n (%)**		
Atrial fibrillation†	14 (42%)	10 (31%)
Arrhythmias other than AF	4 (12%)	0
Low cardiac output syndrome	10 (30%)	8 (25%)
Pleural effusion	4 (12%)	5 (16%)
Stroke	0	1 (3%)
Myocardial Infarction	0	0
Heart failure	0	2 (6%)
Infection	4 (12%)	1 (3%)
Acute kidney injury	3 (9%)	2 (6%)
Death	0	0

* $p > 0.05$ Atorvastatin vs Placebo, by Fisher's exact test.

† AF diagnosed by continuous Holter ECG recordings over 5 postoperative days.

Supplementary Table 4 STARR - Measurement of atrial effective refractory period

A

Number of patients without AERP measurements	**38**
**Reasons**	
Equipment availability	**11**
Post-operative atrial fibrillation	**21**
Pacing wire dislodgement and non-capture	**2**
Complete heart block after aortic valve replacement	**2**
Withdrew consent	**2**

**B***	**Placebo**			**Atorvastatin**		
**Groups**	**PCL 500**	**PCL 600**	**PCL 700**	**PCL 500**	**PCL 600**	**PCL 700**
POD2 vs POD1	0.90	0.92	0.91	0.84	0.77	0.85
POD3 vs POD2	0.89	0.88	0.88	0.84	0.66	0.53
POD4 vs POD3	0.86	0.90	0.82	0.89	0.99	0.90

**C†**	**Placebo**	**Atorvastatin**	**P value**
**PCL**	**Slope (95%C.I)**	**Slope (95%C.I)**	
500	1.5 (-0.4 to 3.3)	1.9 (0.1 to 3.7)	0.72
600	3.1 (1.1 to 5.2)	1.8 (-0.3 to 3.8)	0.36
700	2.1 (-0.02 to 4.3)	3.6 (1.2 to 6.0)	0.38

* Non parametric spearman’s correlation matrix. † Data are shown after log transformation. AERP, Atrial effective refractory period; PCL, Pacing cycle length; POD, Postoperative day.

1. Supplementary methods

1.1 Measurement of myocardial superoxide production

Superoxide production was measured in atrial tissue homogenates by two different methods: lucigenin (5 µmol/L)-enhanced chemiluminescence and 2-hydroxyethidium (2-OH E) detection by high-performance liquid chromatography (HPLC), as described previously[1], with results shown as the tiron-inhibitable fraction. To elucidate the enzymatic sources of atrial superoxide production, aliquots of atrial homogenates were pre-treated with: gp91-ds-tat peptide (10 µM/L) to inhibit NOX2 oxidases [2] and the respective scrambled peptide as negative control; and Rotenone (100 µMol/L) to inhibit mitochondrial complex I.

1.2 Measurement of myocardial peroxynitrite

The uric acid-inhibitable fraction of luminol chemiluminescence was taken as measurement of peroxynitrite (ONOO-) content in atrial homogenates. Luminol (100 µmol/L) was used as the chemiluminescent probe and uric acid (1 mmol/L) as peroxynitrite scavenger [3].

1.3 Measurement of myocardial NOS activity

NOS activity was measured in atrial tissue homogenates using radiochemical HPLC detection of ^{14}C labelled L- arginine to L-citrulline conversion and expressed as the L- NAME inhibitable fraction, as described previously[4].

1.4 Quantification of myocardial biopterins

Tetrahydrobiopterin (BH_4), and its oxidized products 7,8 dihydro biopterin (BH_2) and biopterin (B) were measured by electrochemical (for BH_4) and fluorescence detection

(for BH_2 and B) in homogenized atrial tissue, following sample separation by HPLC, as described previously[1].

1.5 Measurement of myocardial GTPCH-1 activity

The activity of the rate-limiting enzyme in the synthesis of BH_4, GTP cyclohydrolase 1 (GTPCH) was measured in atrial homogenates by iodine oxidation and detection of neopterin content by HPLC, as described previously[4].

1.6 Immunoblotting

For immunoblotting, primary antibodies raised against nNOS (Santa Cruz,1/2000), iNOS (Millipore1/1000), eNOS (Santa Cruz, 1/4000), GTPCH (Abnova,1/500), GTP cyclohydrolase feedback regulatory protein (Cell signalling, 1/250), NOX2 (BD Biosciences, 1/1000) and NOX4 (AbCam, 1/1000) were used. Immunodetection of primary antibodies was performed using horseradish peroxidase-conjugated secondary antibodies (Promega, USA). Bands were visualized with enhanced chemifluorescence (Amersham Bioscience UK Ltd.) and imaged using the Bio-Rad ChemiDoc XRS system. The 2D densities of the bands were quantified using the Image J program (NIH) and normalised to either beta-Tubulin (Santa Cruz, 1/1000) or GAPDH (Sigma, 1/1000). To re-probe with a different primary antibody, membranes were stripped using Re-blot plus (Millipore Inc), as per manufacturer's recommendations.

1.7 Rac1 activation assay

Rac1 activity, defined by the ratio of GTP-Rac1 to total Rac1, was evaluated by a commercially available affinity precipitation assay with p21-activated kinase (PAK)-1 fusion protein conjugated glutathione agarose beads (PAK1-PBD) according to the

manufacturer's protocol (Millipore, Temecula, California, USA), as described previously[1].

1.8 Blood assays

All blood assays were carried out by the CTSU's Wolfson Laboratories, University of Oxford, blind to the study treatment allocation. Plasma and serum were separated by centrifugation at 1300g for 10 minutes at room temperature. For the evaluation of LDL-C, Troponin I and Creatinine, Beckman Coulter ACCESS 2 (Chemiluminescent immunoassay; Troponin) and Beckman Coulter AU680 (Endpoint Assay for LDL and Jaffé Method A for Creatinine) were used as per the manufacturer's recommendations. For the evaluation of NT pro BNP, the Meso scale discovery (MSD) sector Imager 6000 (Electrochemiluminescence Assay) platform was used. Assays were validated to confirm the accuracy, precision, linearity, recovery and reportable range. In addition, stability of troponin on room temperature was assessed prior to the analysis.

References

1. Reilly SN, Jayaram R, Nahar K, Antoniades C, Verheule S, Channon KM, Alp NJ, Schotten U, Casadei B. Atrial sources of reactive oxygen species vary with the duration and substrate of atrial fibrillation: implications for the antiarrhythmic effect of statins. *Circulation* 2011;**124**:1107-1117.
2. Rey FE, Cifuentes ME, Kiarash A, Quinn MT, Pagano PJ. Novel Competitive Inhibitor of NAD(P)H Oxidase Assembly Attenuates Vascular O2- and Systolic Blood Pressure in Mice. *Circ Res* 2001;**89**:408-414.
3. Guzik TJ, Channon KM. Measurement of Vascular Reactive Oxygen Species Production by Chemiluminescence. In: Fennell JP, Baker AH, eds. Hypertension: Methods and Protocols. Totowa, NJ: Humana Press, 2005:73-89.

4. Carnicer R, Hale AB, Suffredini S, Liu X, Reilly S, Zhang MH, Surdo NC, Bendall JK, Crabtree MJ, Lim GB, Alp NJ, Channon KM, Casadei B. Cardiomyocyte GTP cyclohydrolase 1 and tetrahydrobiopterin increase NOS1 activity and accelerate myocardial relaxation. *Circ Res* 2012;**111**:718-727.

UNCROPPED GELS

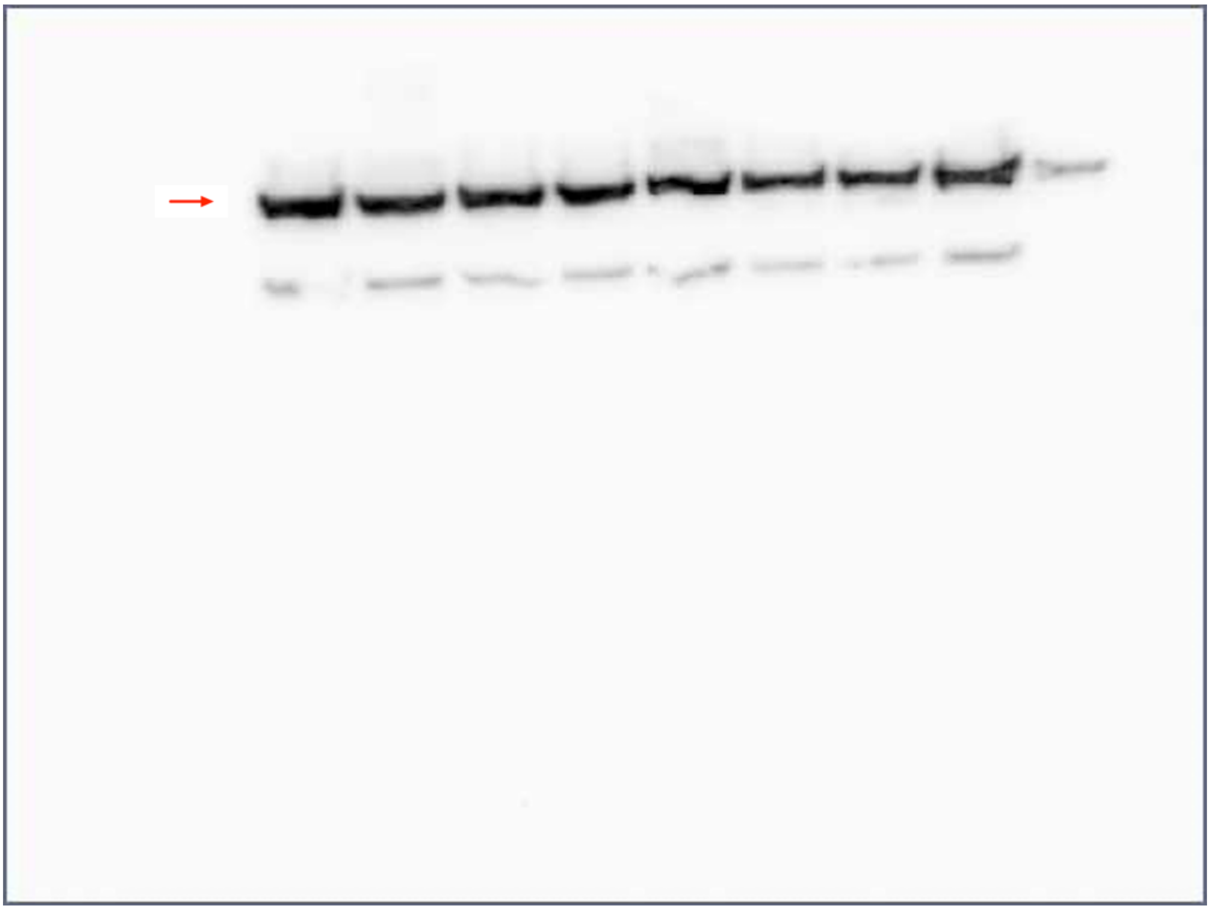

Figure 2B_Beta Tubulin protein

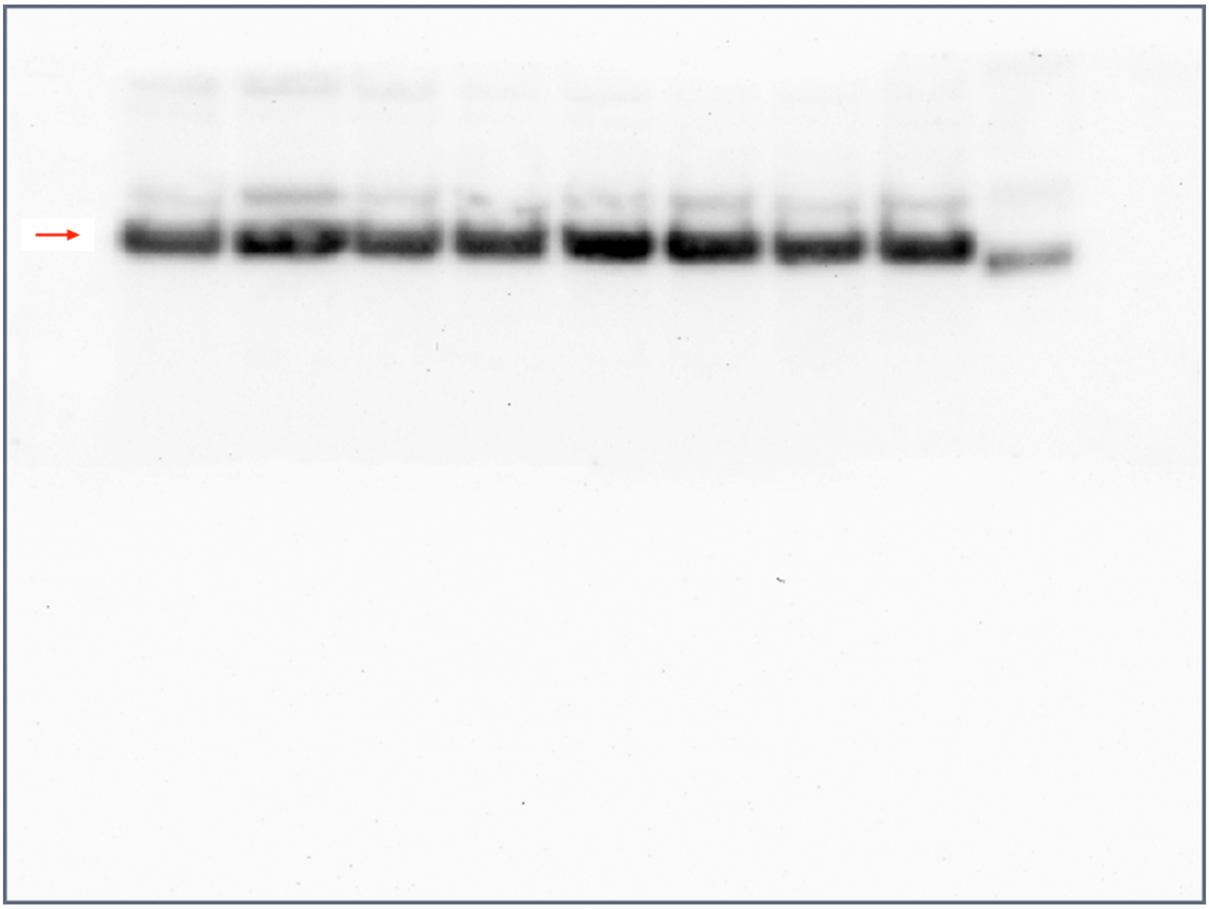

Figure 2B_nNOS protein

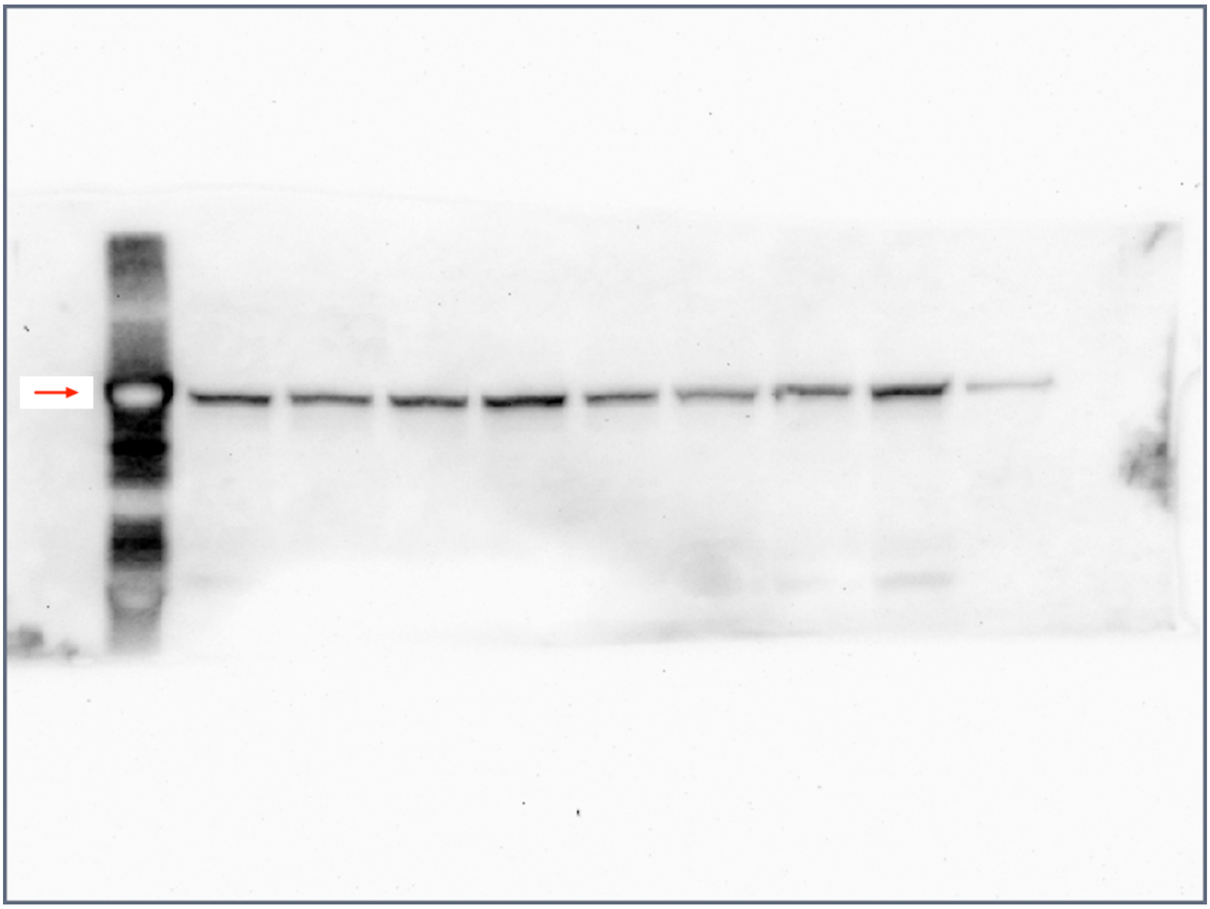

Figure 2C_eNOS protein

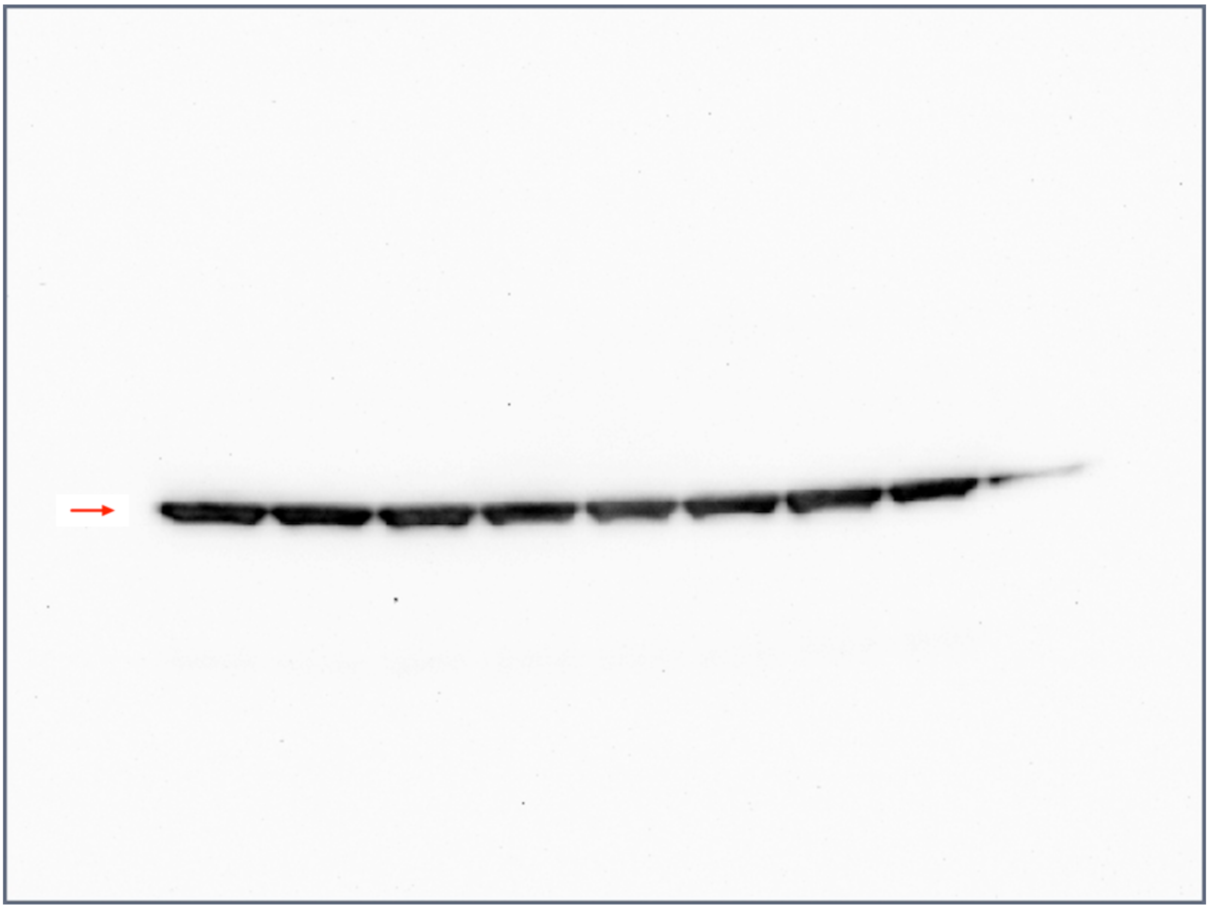

Figure 2C_GAPDH protein

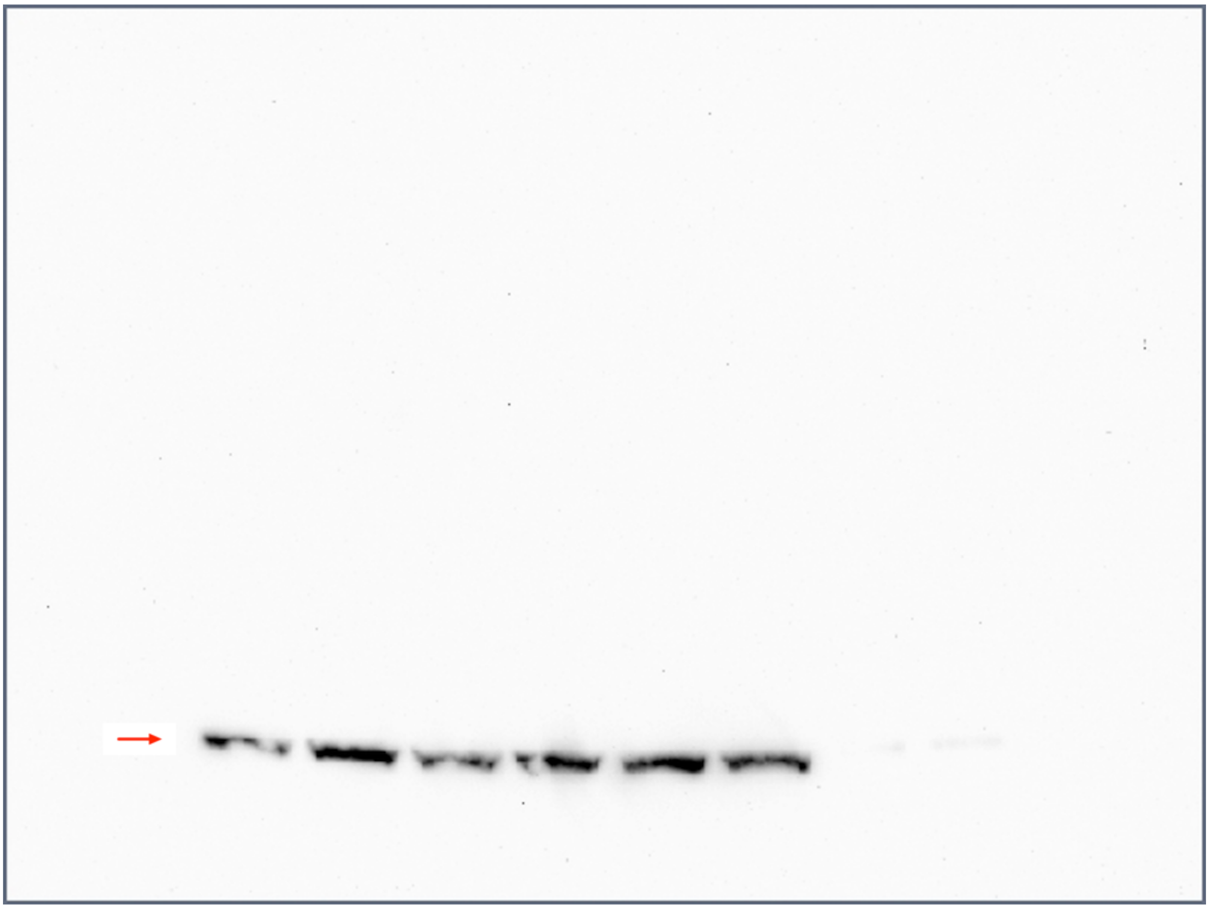

Figure 2D_ Beta Tubulin protein

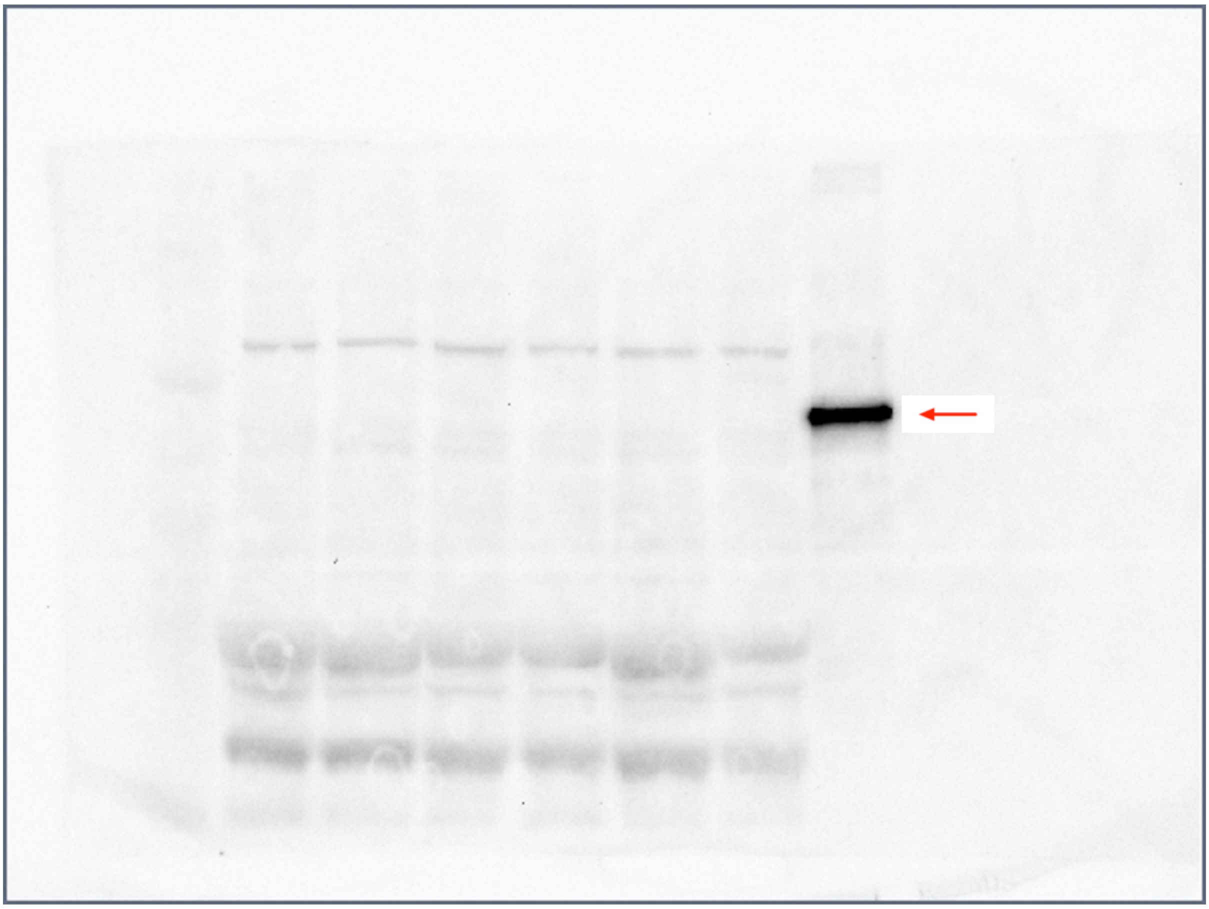

Figure 2D_iNOS protein

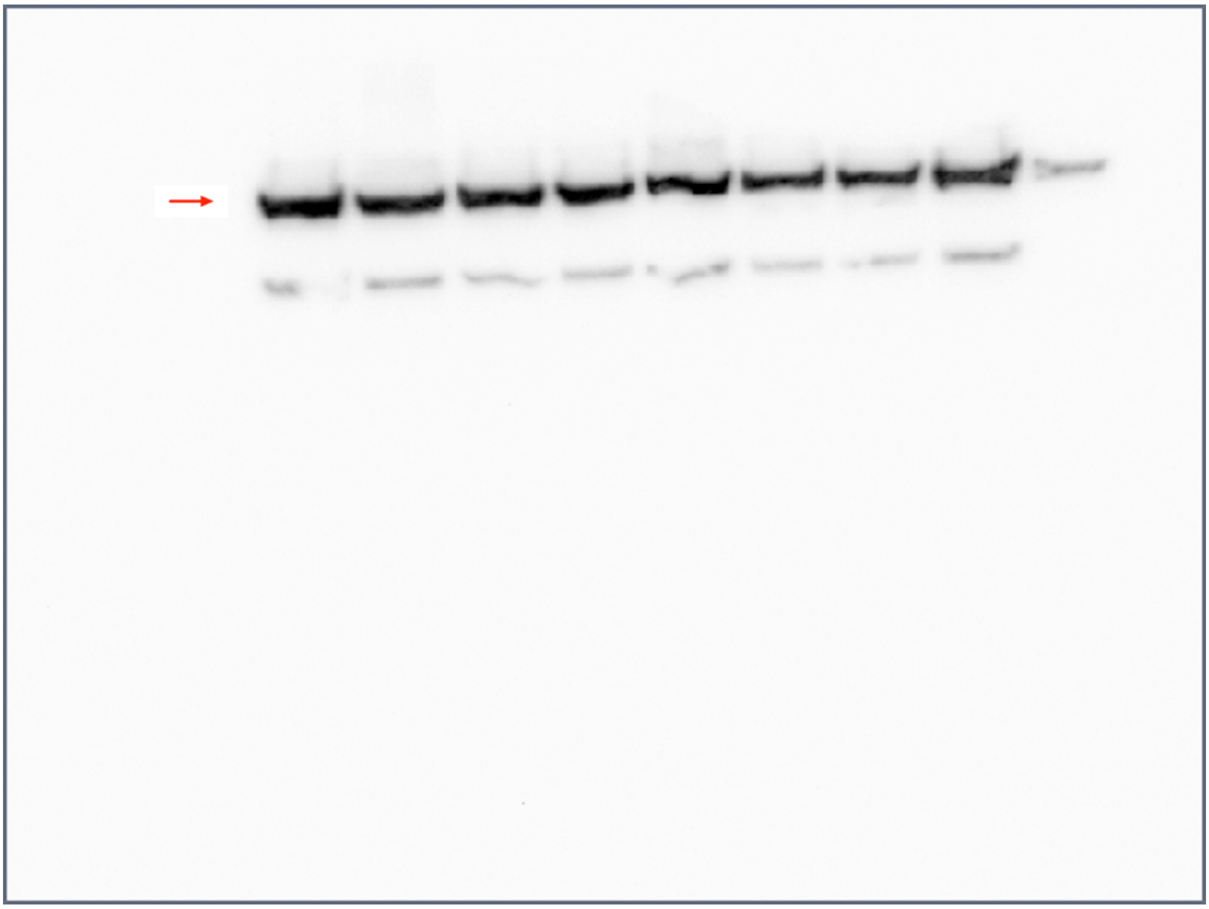

Figure 4 B_ Beta Tubulin protein

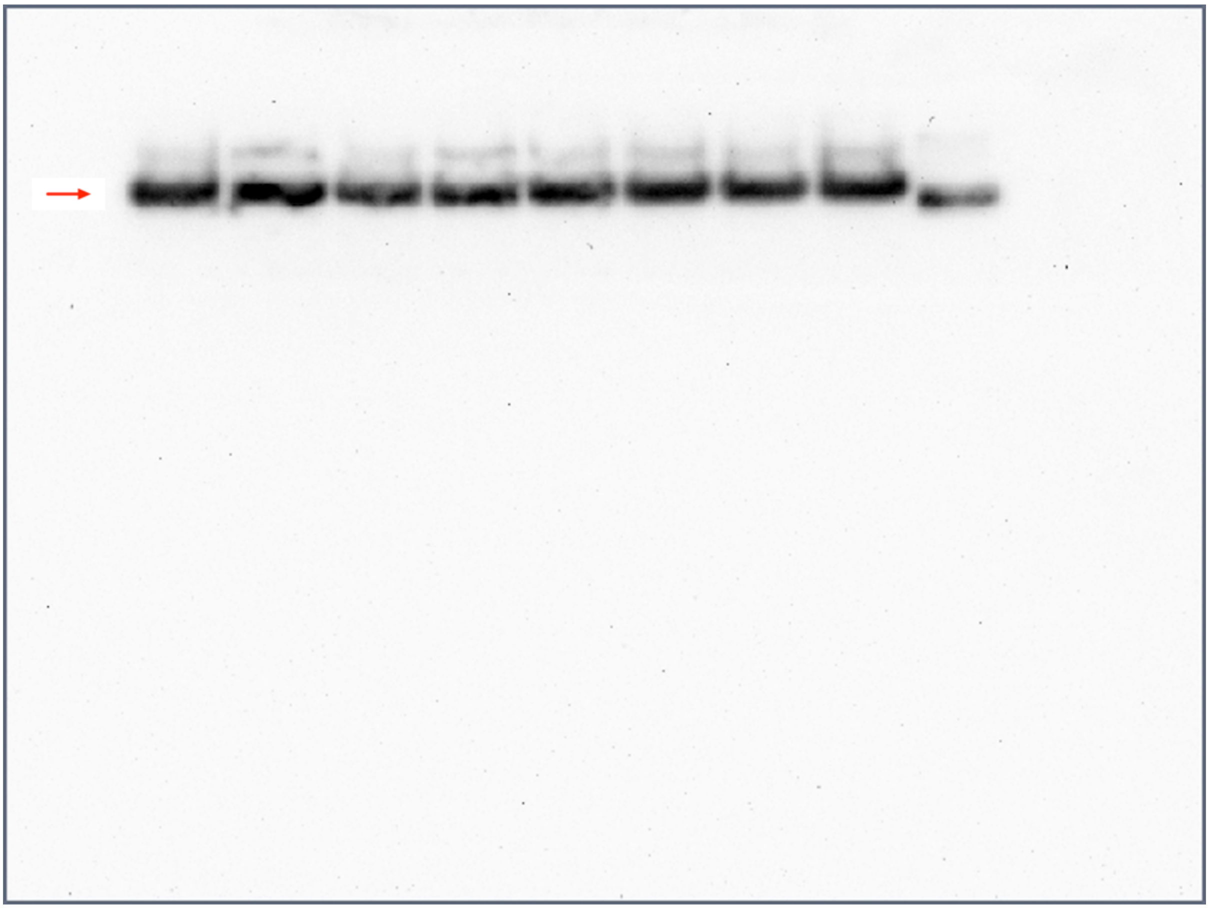

Figure 4 B_GCH protein

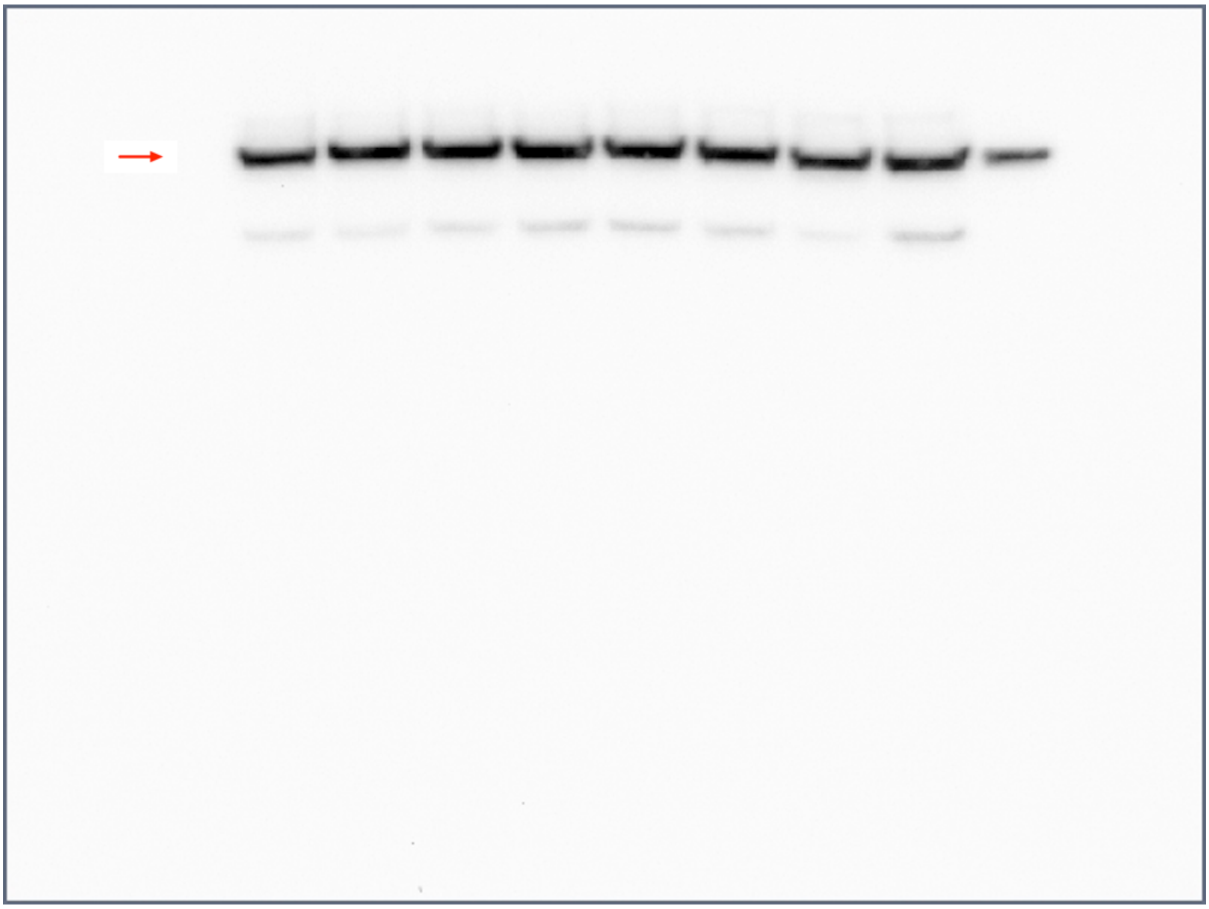

Figure 4 C_ Beta Tubulin protein

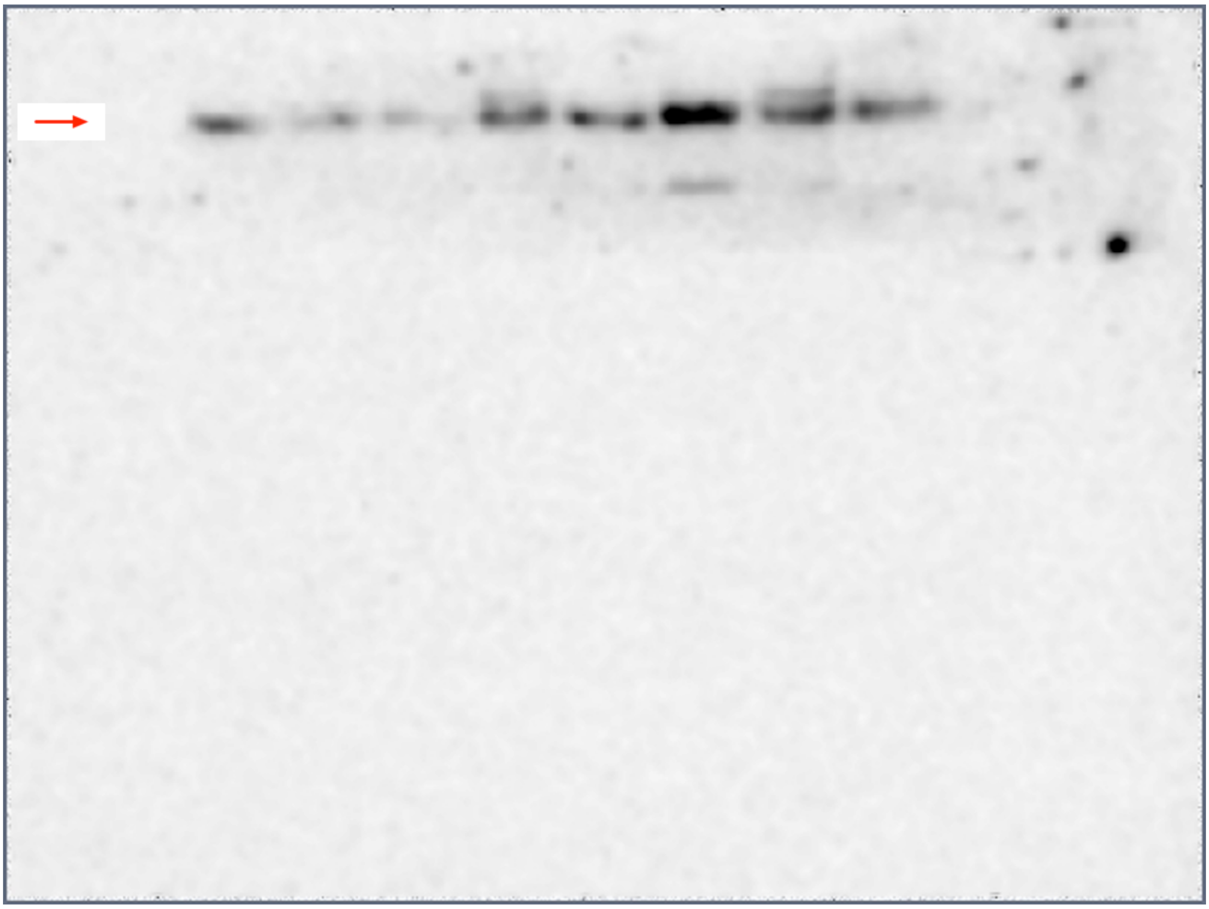

Figure 4 C_GFRP Protein

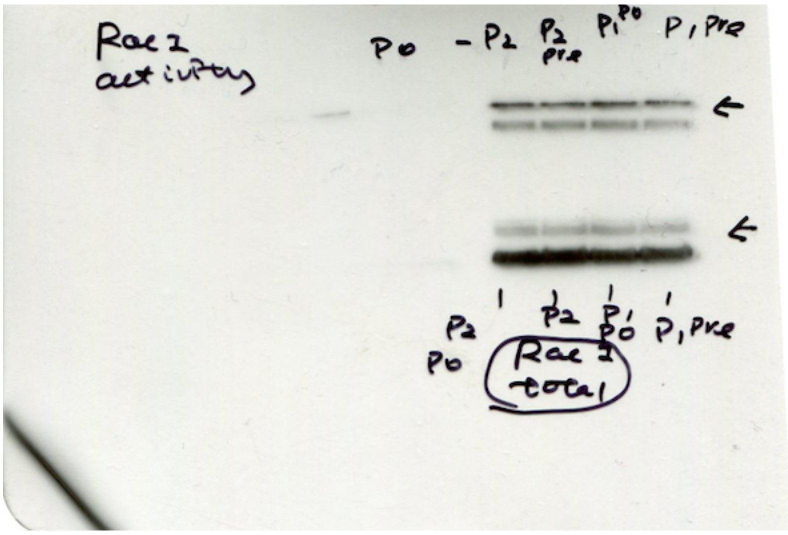

Supplementary Figure 5 A_GTP_RAC-1/Total RAC-1 protein

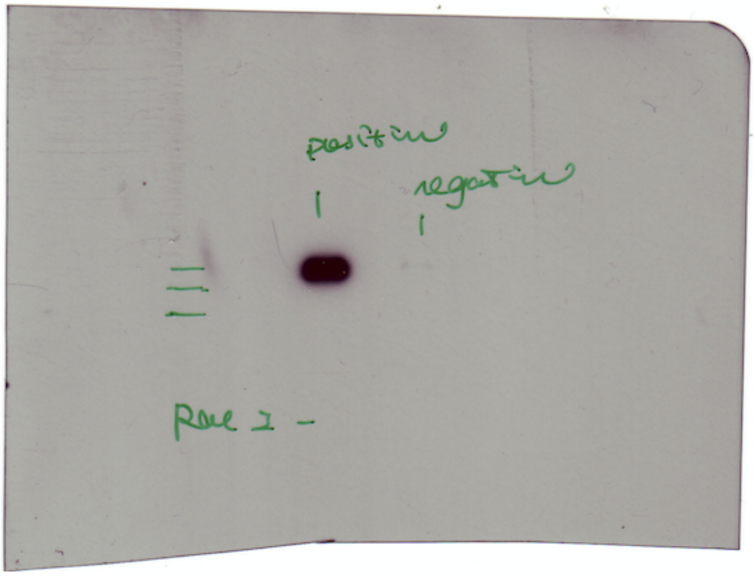

Supplementary Figure 5 A_GTP-RAC-1 + and – controls

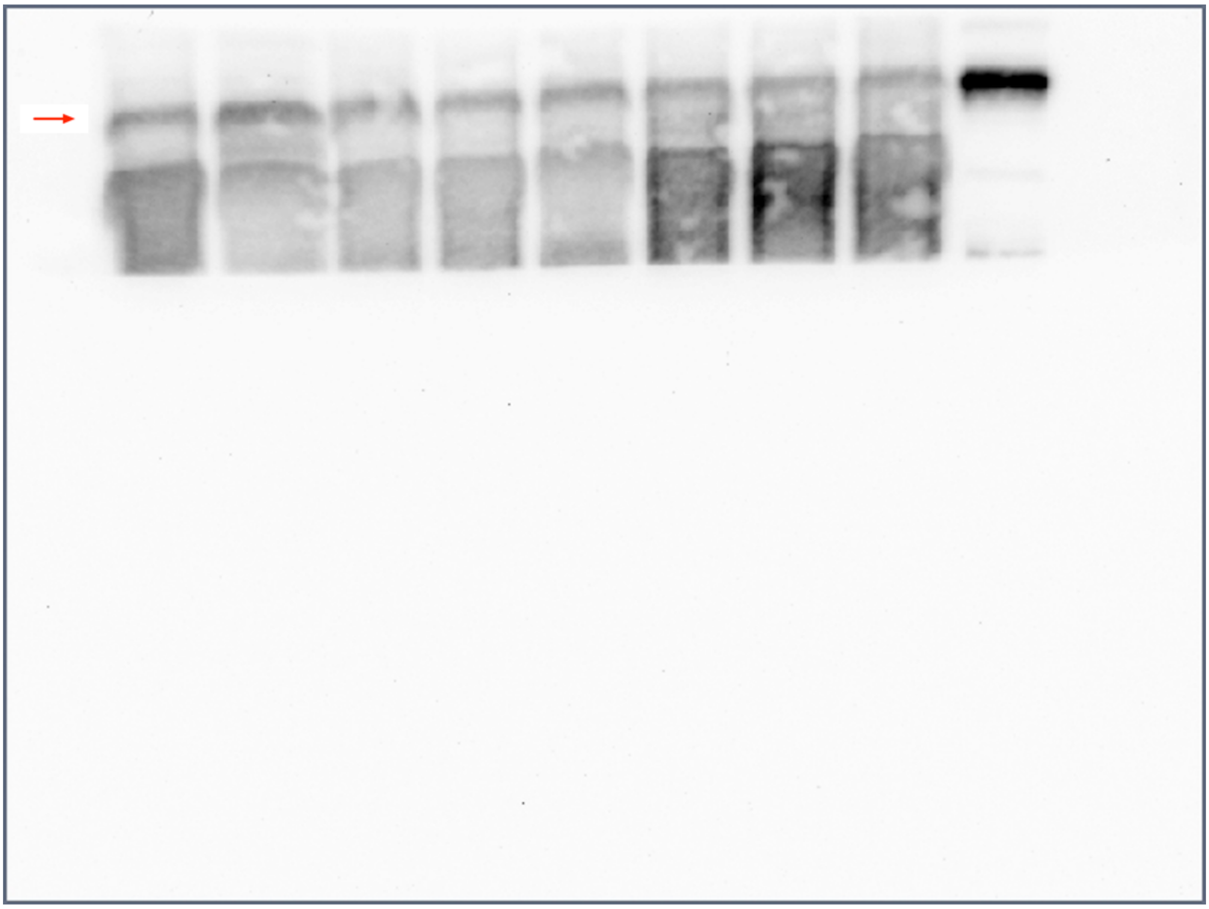

Supplementary Figure 5 B_NOX2 protein

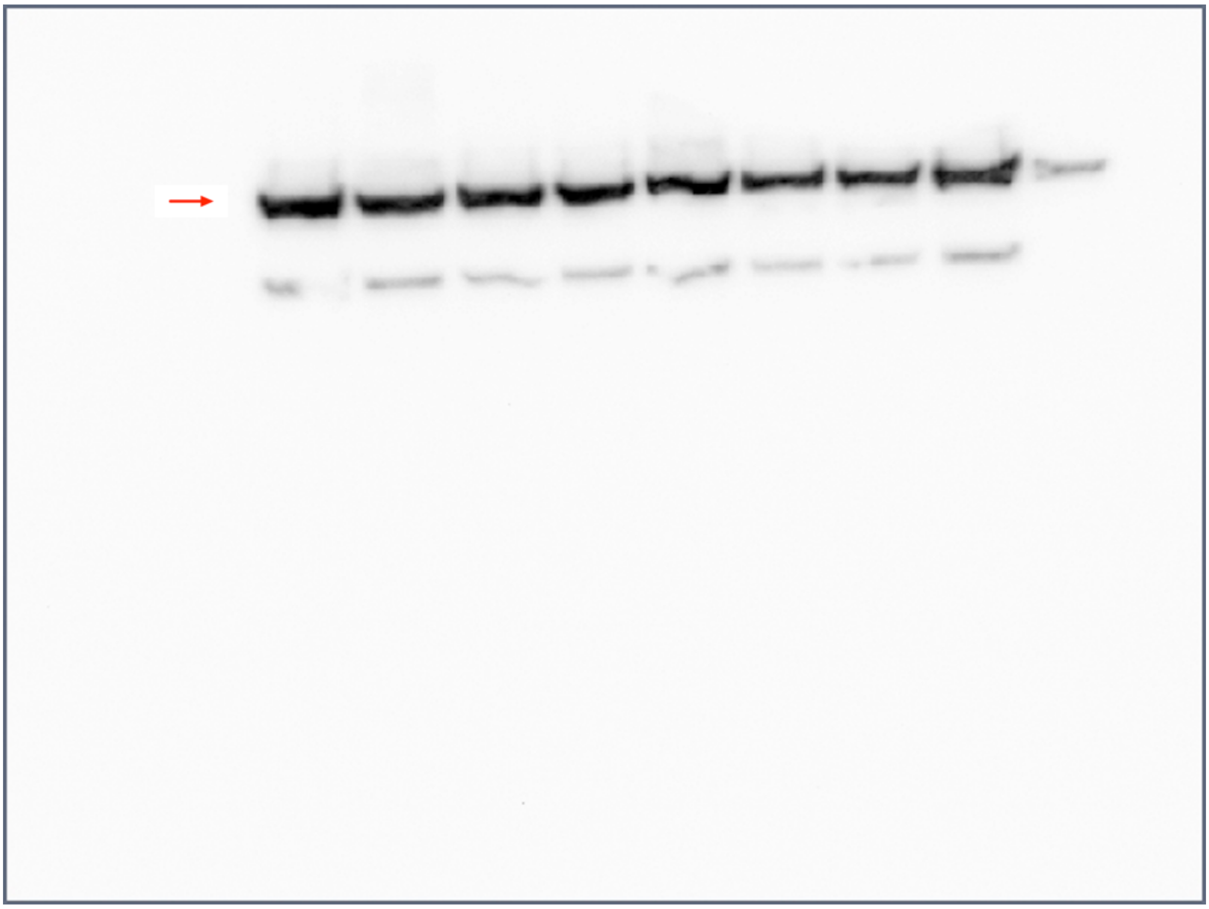

Supplementary Figure 5 B_Beta Tubulin protein

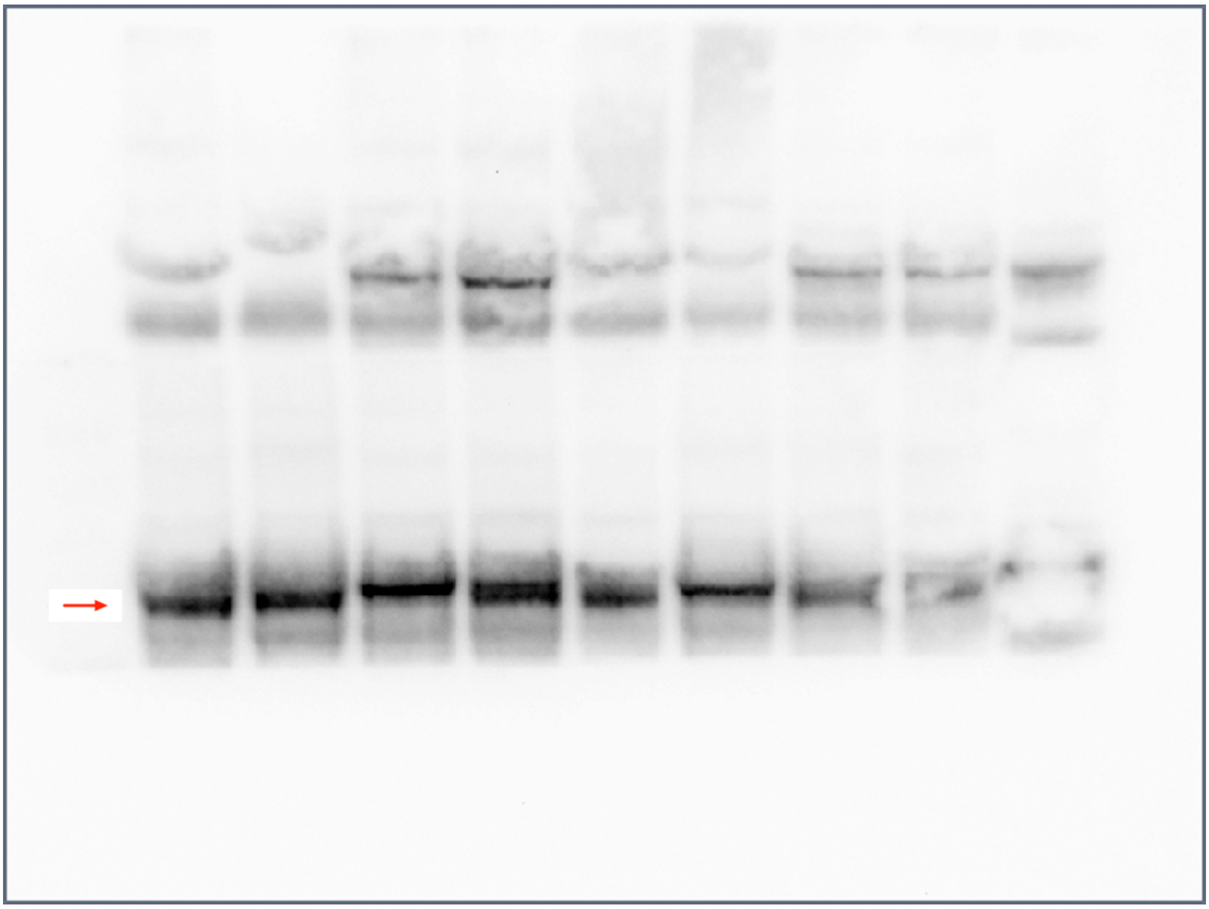

Supplementary Figure 5 C_NOX4 protein

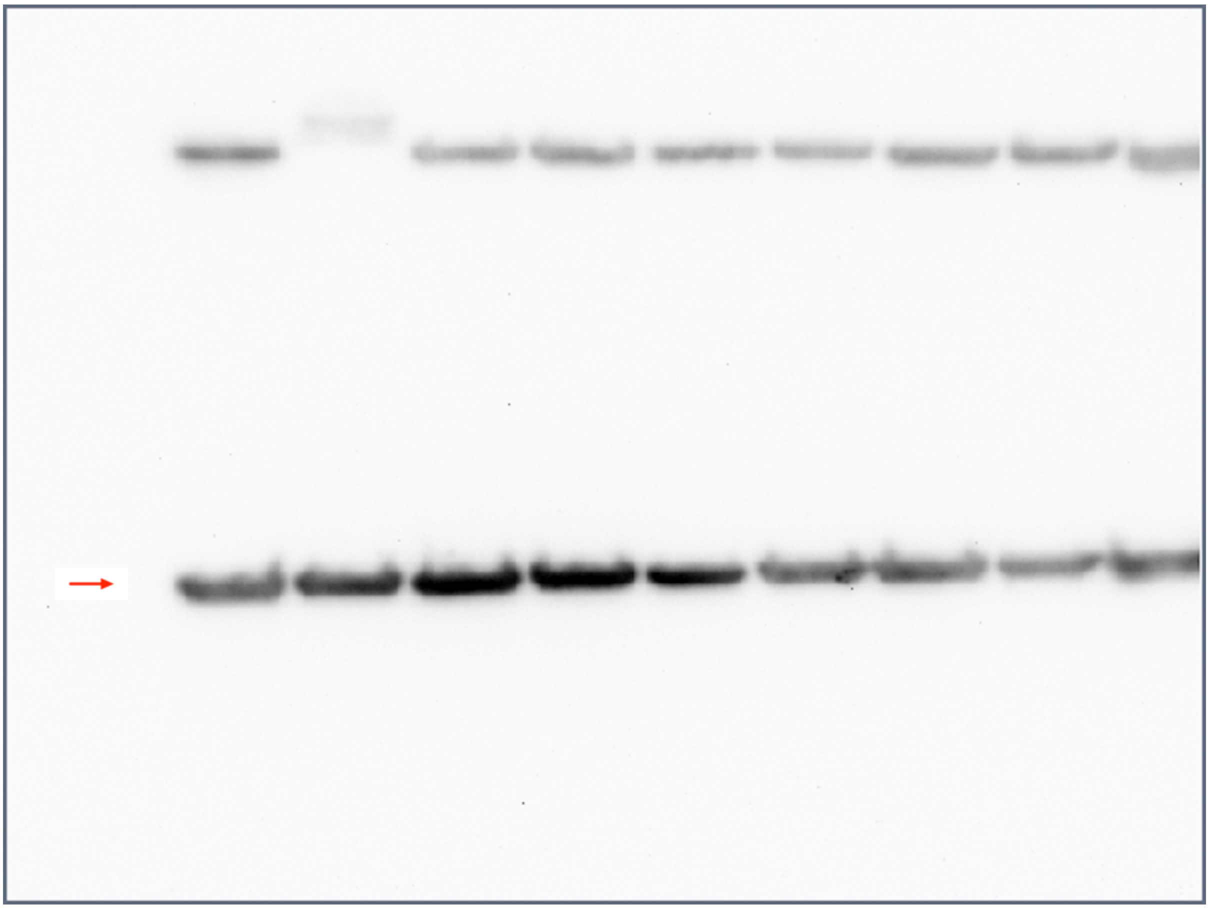

Supplementary Figure 5 C_GAPDH protein
